# Retina as a window to cerebral dysfunction following studies with circRNA signature during neurodegeneration

**DOI:** 10.7150/thno.51550

**Published:** 2021-01-01

**Authors:** Qin Jiang, Dong-Yuan Su, Zhen-Zhen Wang, Chang Liu, Ya-Nan Sun, Hong Cheng, Xiu-Miao Li, Biao Yan

**Affiliations:** 1 The Affiliated Eye Hospital, Nanjing Medical University, Nanjing, China.; 2 The Fourth School of Clinical Medicine, Nanjing Medical University, Nanjing, China.; 3 Jiangsu Province Key Laboratory of Neurodegeneration, Center for Global Health, Nanjing Medical University, Nanjing, China.; 4 Eye Institute, Eye & ENT Hospital, Shanghai Medical College, Fudan University, Shanghai, China.; 5 Department of Neurology, Jiangsu Province Hospital, Nanjing, China.; 6 NHC Key Laboratory of Myopia (Fudan University), Key Laboratory of Myopia, Chinese Academy of Medical Sciences, and Shanghai Key Laboratory of Visual Impairment and Restoration (Fudan University), Shanghai, China.

**Keywords:** Circular RNAs, Ischemia, MCAO, OGD/R, retinal neurodegeneration

## Abstract

Ischemia-induced cerebral injury is a major cause of dementia or death worldwide. The pre-diagnosis is still challenging due to the retarded symptoms. The retina is regarded as the extension of cerebral tissue. Circular RNAs have emerged as the crucial regulators in gene regulatory network and disease progression. However, it is still unknown whether circRNAs can be used as the common regulators and diagnostic markers for cerebral neurodegeneration and retinal neurodegeneration.

**Methods:** C57BL/6J mice were subjected to transient middle cerebral artery occlusion and circRNA microarray profiling was performed to identify neurodegeneration-related circRNAs. Quantitative reverse-transcription PCR (qRT-PCR) assays were performed to verify circRNA expression pattern. Gene ontology (GO) and Kyoto Encyclopedia of Genes and Genomes (KEGG) pathway analysis was performed to determine the biologic modules and signaling pathway. TTC staining, Nissl's staining, and immunofluorescence staining assays were performed to investigate the role of circRNA in cerebral neurodegeneration and retinal neurodegeneration *in vivo*. MTT assay, Propidium iodide (PI)/Calcein-AM staining, and Rhodamine 123 assays were performed to investigate the role of circRNA in neuronal injury *in vitro*. Bioinformatics, RIP, and luciferase activity assays were performed to determine the regulatory mechanism of circRNA in neurodegeneration.

**Results:** 217 differentially expressed circRNAs were identified between ischemic cerebral tissues and normal controls. Among them, cGLIS3 was shown as the common regulator of cerebral neurodegeneration and retinal neurodegeneration. cGLIS3 silencing alleviated ischemia-induced retinal neurodegeneration and MCAO-induced cerebral neurodegeneration *in vivo*. cGLIS3 silencing protected against OGD/R-induced RGC injury *in vitro*. The circulating levels of cGLIS3 were significantly increased in the patients with ischemic stroke compared to healthy subjects. cGLIS3 levels were also increased in the aqueous humor of the patients with retinal vein occlusion. cGLIS3 regulated neuronal cell injury by acting as miR-203 sponge and its level was controlled by EIF4A3.

**Conclusions:** This study provides molecular evidence that the retina is window of the brain from circRNA perspective. cGLIS3 is a common regulator and diagnostic marker of cerebral neurodegeneration and retinal neurodegeneration.

## Introduction

Ischemia is a common pathological factor in a series of neurodegenerative diseases, including ischemic retinopathy, stroke, cerebral embolism, and Alzheimer's disease 1, 2. The patients with ischemic complications have impaired quality of life. Ischemia-induced neurodegenerative diseases have become the major causes of death and severe disability among adults worldwide 3. Ischemia caused by inadequate blood supply can lead to serious tissue injury. The neurons and glia are vulnerable to ischemia-induced injury. Long-term ischemia may induce irreversible cell death 4. The mechanisms responsible for ischemic neurodegeneration mainly include energy failure, mitochondrial dysregulation, increased oxidative stress, generation of free radicals, and neuroinflammation 5.

The pathogenesis of ischemia is highly complicated and regulated by at transcriptional, post-transcriptional, epigenetic, translational, or post-translational level 6, 7. Circular RNAs (circRNAs) are a group of endogenous non-coding RNAs characterized by the covalently closed cyclic structures 8. They have attracted attention due to their versatile roles in regulating gene expression by acting as miRNA sponge, transcription regulator, or protein sponge 9. circRNAs are more stable than the liner non-coding RNAs because they can resist the degradation of RNA exonucleases 10, 11. Previous studies have revealed that circRNAs participate in many developmental and physiological processes, such as cell proliferation, apoptosis, and differentiation 11. circRNAs tend to be aberrantly expressed and are regarded as the potential biomarkers in several human diseases, such as cancers, cardiovascular diseases, and neurological diseases 12.

Ischemic stroke is an ischemia-induced neurodegenerative disease in central nervous system 13. Nowadays, stroke diagnosis is still based on clinical criteria and imaging data according to the occurrence of tissue lesions. At this point, the disease has progressed to the advanced or late stage. The treatment for stroke is mainly through the early reperfusion and/or mechanical recanalization of obstructed arteries 14. However, the efficacy is limited by the narrow time window, risk of bleeding, and other factors. Ischemic stroke is also associated with concomitant vision impairment and loss 15. Generally, the retina is recognized as the extension of the brain. The retina and the brain may share the common regulators and have similar neurodegenerative changes. Moreover, the ocular symptoms sometimes precede the appearance of stroke symptoms 16-18. Hence, the accessibility and organization of the retina makes it a convenient and non-invasive tool for stroke research.

circRNAs have shown great potentials as the biomarkers and targets for disease diagnosis and prognosis. The role of circRNAs in stroke have been recognized. circDLGAP4 can ameliorate ischemic stroke outcomes via regulating endothelial-mesenchymal transition 19. circTLK1 can aggravate neuronal injury and neurological deficits after ischemic stroke via miR-335-3p/TIPARP 20. The roles of circRNAs in retinal diseases have also been recognized. circZNF609 regulates retinal neurodegeneration by acting as miR-615 sponge 21. circZRANB1 is a promising target for the therapy of retinal neurodegeneration 22. However, it is still unknown whether cerebral neurodegeneration and retinal neurodegeneration is regulated by the common circRNA. In this study, we used two different models, ischemic stroke and ischemic retinopathy, to investigate the expression profiling and functional significance of circRNAs in neurodegeneration. We also determined whether the retina and the brain shared the common circRNA regulator and the common circRNA disease marker.

## Materials and Methods

### Animal experiments

The experiments were performed under the guidelines of the ARVO Statement for the Use of Animals in Ophthalmic and Vision Research and approved by the Animal Care and Use Committee of Nanjing Medical University. C57BL/6 mice (male, 25-30 g) were purchased from Nanjing Qinglongshan Experimental Animal Center (Nanjing, China) and kept under the pathogen-free condition. They were maintained at room temperature, humidity of 50% ± 10%, and alternating 12 h light/dark cycle.

### Middle cerebral artery occlusion (MCAO) model

C57BL/6 mice (male, weighing 25-30 g) were anesthetized with 2.5% isofurane (RWD Life Science, China) and maintained in 2.5% isoflurane with 70% N_2_ and 30% O_2_. Under an operating microscope, a midline neck incision was made with scissors between the manubrium and the jaw. The right common carotid artery (CCA), external carotid artery (ECA), internal carotid artery (ICA), and vagus nerve were exposed. The filament was introduced into the right internal carotid artery up to the anterior cerebral artery to occlude the MCA and the anterior choroideal arteries. After the operation, the surgical site was sutured and re-perfused. Rectal temperature was maintained at 37 ± 0.5 °C with an electric blanket. For the sham groups, all procedures were identical except that the occluding monofilament was not inserted.

### circRNA microarray profiling

Total RNAs were isolated from 9 ischemic cortex or 9 non-ischemic cortex using TRIzol reagent (Life Technologies, CA, USA) at 1 week after surgery. Three samples were pooled together as a biological replicate to reduce biological variation. The concentration and quality of RNA samples were determined using the NanoDrop ND 1000 spectrophotometer (Thermo Fisher Scientific, Waltham, MA, USA). CircRNAs were enriched by removing the linear RNAs with RNase R (Epicentre Inc. USA), amplified, and transcribed into the fluorescent cRNAs (Super RNA Labeling Kit, Arraystar). The fluorophore-labeled cRNAs were purified using the RNeasy Mini Kit (Qiagen). Gene Expression Hybridization Kit (Agilent Technologies) was used for hybridization. An Agilent microarray scanner was used to obtain circRNA microarray signal. The microarray signal was extracted using the Agilent Feature Extraction software 10.7 (Agilent technologies). Raw data summarization, normalization, and quality control was performed using the Agilent GeneSpring version 13.0 (Agilent Technologies). Differentially expressed circRNAs were identified by volcano plot filtering. circRNA expression patterns across different samples were shown through hierarchical clustering.

### Quantitative real-time PCR

Total RNAs were reversely transcribed into a final volume of 20 μl cDNAs using the Reverse master mix regent Kit (Takara). Quantitative PCR assays were determined using SYBR Premix Ex Taq (Takara) in the PikoReal Real-Time PCR System (Thermo Scientific, USA). qPCR reactions were performed at the following conditions: initial denaturation at 94 °C for 2 min, 50 cycles; denaturation at 94 °C for 10 s; and annealing and elongation at 60 °C for 20 s. The relative expression change of each circRNA was calculated by the cycle threshold method (2^-ΔΔCt^ method).

### Gene ontology and KEGG pathway analysis of selected circRNAs

To investigate the potential functions of the host genes of selected circRNAs, these genes were subjected for Gene Ontology (GO) and Kyoto Encyclopedia of Genes and Genomes (KEGG) pathway analysis. GO enrichment analysis was performed to reveal biological process (BP), molecular function (MF), and cellular component (CC). KEGG pathway analysis was performed to reveal potential signaling pathways.

### TTC staining and infarct volume calculation

The mice were euthanized at 24 h after reperfusion. Briefly, the cerebral tissues were dissected and frozen at -20 °C for 30 min. Beginning 1 mm posterior to the anterior pole, the cerebral tissue was sliced into 4 serial coronal sections (2 mm thick). The slices were stained with 2% 2, 3, 5-triphenyltetrazolium chloride (TTC; Sigma, St. Louis, MO, USA) for 15 min at 37 °C in dark to differentiate between ischemic region and non-ischemic region. After rinsing with PBS, the slices were fixed in 4% PFA for 1.5 h. The stained sections were scanned after 12 h and the infarct volume was determined by image analysis.

### Neurological severity score (NSS) test

NSS test was performed to evaluate the degree of neurological deficits using the modified Bederson scoring method. Briefly, the mice were evaluated and given deficit scores according to the following parameters: if mice had no apparent deficits, the mice were given a score of 0. If the presence of forelimb flexion, decreased resistance to push, or apparent circling, the mice were given a score of 1, 2, or 3, respectively.

### Nissl staining

After dehydration with gradient ethanol elution and cleared in xylene, cerebral tissues were sliced into 10 μm coronal sections using a cryostat (Leica CM1950, Leica) and stored at -20 °C. Nissl staining (Beyotime Institute of Biotechnology, Haimen, China) was conducted according to the manufacturer's instructions. In brief, the sections were soaked in 1% toluidine blue for 5 min at 50 °C, washed by double distilled water, and dehydrated with gradient ethanol elution. The slice sections were observed under a fluorescent microscope (Olympus IX73).

### Immunofluorescence staining

The cerebral cortex and eyeballs were dissected after MCAO treatment. The eyeballs were fixed in Fekete's solution for 1 h. The cornea, lens, and vitreous humor were removed. Then, cortex and retinas were fixed in 4% PFA overnight at 4 °C. Next, they were dehydrated in a gradient sucrose solution (15% and 30%) at 4 °C, followed by embedding in OCT compound. Serial sections of cerebral cortex (20 μm thick) and eye tissue (5 μm thick) were cut using a cryostat (Thermo Scientific, USA) and placed on adhesion microscope slides (Citotest). After washing twice with PBS, the frozen sections were permeabilized and blocked with 5% bovine serum albumin (BSA) for 30 min. Then, they were incubated with the primary antibody overnight at 4 °C and washed with PBST three times. The slides were incubated with the secondary antibody for 2 h and 4, 6-diamidino-2-phenylindole (DAPI, 1:1500, Beyotime, C1002) for 10 min in dark. The sections were observed under a fluorescent microscope (Olympus IX73).

### Cell culture and transfection

Retinal ganglion cells (RGCs) were cultured in high-glucose Dulbecco's modified eagle medium (DMEM, Gibco, USA) supplemented with 10% fetal bovine serum (FBS, Gibco) and 1% penicillin/streptomycin (Invitrogen, USA). RGCs were maintained in a humidified incubator that contained 95% air/5% CO_2_ at 37 °C. cGLIS3 silencing was achieved using the specific siRNA (GenePharma, China). One day prior to transfection, RGCs were seeded into 24-well plates to reach approximately 70% confluence. The lipofectamine 6000 (Beyotime, C0526) transfection reagent were used for cGLIS3 silencing.

### Oxygen-glucose deprivation/reoxygenation (OGD/R) model

OGD/R model was established to mimic ischemic injury *in vitro*. After the required treatment, RGCs were cultured in DMEM medium with the deoxygenated glucose-free Hanks' Balanced Salt Solution (Invitrogen). After 60 min of incubation, these cells were transferred to the hypoxic chamber with mixed gas (0.2% O_2_, 94.8% N_2_, and 5% CO_2_) at 37 °C. After 2 h, the cultures were subjected to reoxytenation by exchanging the medium to glucose restoration and maintained under normoxic conditions for 24 h, respectively. As the control group, these cells were grown in the full media containing glucose under normal conditions.

### Collection of clinical samples

For the collection of aqueous humor, the participants were divided into two groups: the patients were diagnosed with central retinal vein occlusion who underwent intravitreal anti-VEGF injection and the patients with cataract surgery. The included criteria for central retinal vein occlusion are shown below: clinically detectable macular edema for more than 1 month, central subfield macular thickness (CSMT) of 300 μm or greater on spectral-domain optical coherence tomography (OCT), retinal capillary nonperfusion, and visual field defects. The average RVO history was 2.2 ± 0.5 months. Aqueous humor (AH) was collected under the surgical microscope before cataract surgery and anti-VEGF intravitreal injection. The AH samples were immediately transferred into the sterile tubes and centrifuged at 12,000 g for 5 min before storage at -80 °C until further analysis.

The peripheral blood was collected from the patients with stroke who had not experienced interventional or thrombolysis therapy within 24 h after their admission to the hospital. They had acute focal neurological deficits in combination with diffusion weighted imaging-positive lesions on magnetic resonance imaging, or a new lesion on a delayed CT scan. All subjects with Parkinson's disease, Alzheimer's disease or other non-stroke induced neurological disorders were excluded. Non-stroke controls who received regular physical examinations were recruited as the normal controls. The blood containing 3 U/mL heparin was centrifuged at 3, 000 × g for 10 min at 4 °C. The plasma was carefully collected to avoid contamination by erythrocytes and kept at -80 °C until use.

### Propidium iodide (PI)/Calcein-AM staining

PI/Calcein-AM double stain kit (Shanghai, China) was used to detect cell apoptosis. After the required treatment, RGCs were incubated with Calcein-AM (10 μM, C3099, Invitrogen) and PI (10 μM, P3566, Molecular Probes) at 37 °C for 20 min. The images were captured using a fluorescence microscope (Olympus IX73, Tokyo, Japan). The live cells were observed at 490 nm excitation filter, while the dead cells were observed at 545 nm excitation filter.

### Statistical analysis

Statistical analysis was performed using GraphPad Prism 8 (GraphPad Software, San Diego, CA). For the normally distributed data with equal variance, the significant difference was determined by Student's *t*-test (when two groups were compared) or one-way or two-way ANOVA to test the effect of group (when > 2 groups were compared). For the non-normally distributed data or data with unequal variances, the significant difference was determined by non-parametric Mann-Whitney's *U*-test (when two groups were compared) or Kruskal-Wallis' test followed by post-hoc Bonferroni's test (when > 2 groups were compared). *P* < 0.05 was considered statistically significant.

## Results

### MCAO significantly alters circRNA expression profiles

**Figure 1:**
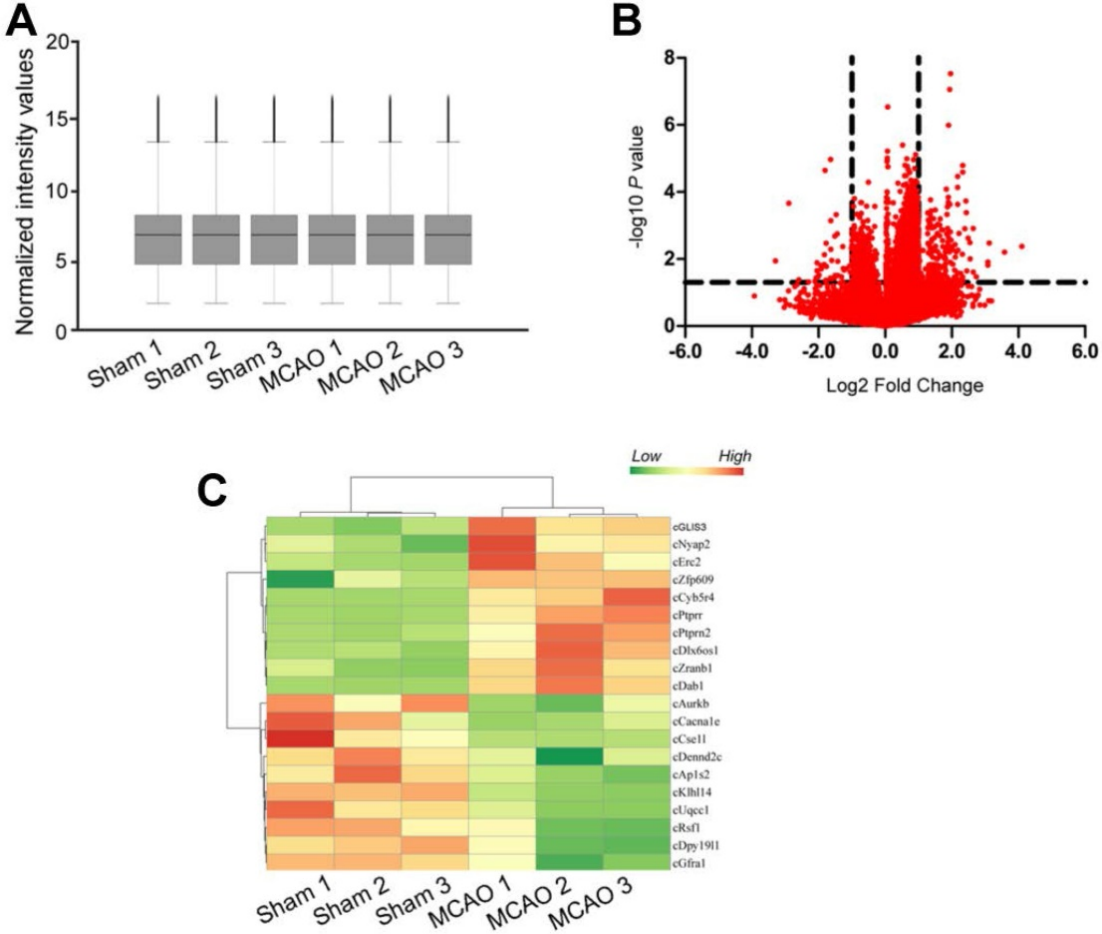
** MCAO significantly alters circRNA expression profiles.** (A) Box plot showed the distribution of circRNA expression across different samples. After the normalization, the distributions of log2 ratios across different samples were nearly the same. The central line was the median of the data, whereas the tail was the upper and lower quartiles. (B) Volcano plot was plotted to show differentially expressed circRNAs between MCAO group and sham-operation group. (C) Hierarchical cluster analysis was used to show the Top 20 differentially expressed circRNAs between MCAO group and sham-operation group. The color scale showed the relative expression of circRNAs across different samples. Up-regulation was shown in orange color and down-regulation was shown in green color.

Focal ischemia was induced by the intraluminal middle cerebral artery occlusion (MCAO) using a 6-0 silicon-coated monofilamentin. Sham-operation group underwent similar surgical procedure except MCAO. Total RNAs were isolated from ischemic group (n = 9) and non-ischemic group (n = 9), respectively. Three samples from the same group were pooled together as a biological replicate to reduce biological variation. circRNA microarrays were performed to determine circRNA expression profiling between MCAO group and sham-operation group. The box plot showed the distributions of circRNA datasets across different biological replicates. The distribution of circRNA expression patterns was nearly the same, suggesting that ischemia-induced changes of individual circRNA levels were not random (Figure 1A). Differentially expressed circRNAs were identified through volcano plot filtering. 217 circRNAs were found to be differentially expressed between MCAO group and sham-operation group (Fold change > 2.0; *P* < 0.05), including 176 up-regulated circRNAs and 41 down-regulated circRNAs (Figure 1B and Table S1). Hierarchical clustering was performed to display circRNA expression patterns between MCAO group and sham-operation group using the top 10 up-regulated circRNAs and the top 10 down-regulated circRNAs. The MCAO samples were clustered together, whereas the sham-operation samples were clustered together (Figure 1C).

### Validation of circRNA expression pattern between MCAO group and sham-operation group by qRT-PCRs

To verify the results of circRNA microarrays, we chosen 20 differentially expressed circRNAs, including 10 up-regulated circRNAs and 10 down-regulated circRNAs in MCAO group. qRT-PCRs were performed to determine the expression pattern of these selected circRNAs. As shown in Table 1, 9 of 10 up-regulated circRNAs and 8 of 10 down-regulated circRNAs had similar expression patterns as the results of circRNA microarrays. Among these circRNAs, cGLIS3 was verified to the most significantly up-regulated circRNA in MCAO group.

### GO and KEGG pathway analysis of the host genes of differentially expressed circRNAs

**Figure 2:**
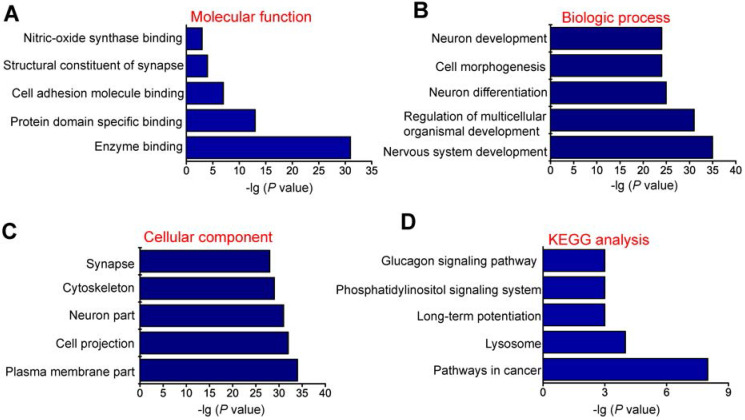
** GO and KEGG pathway analysis of the host genes of dysregulated circRNAs.** GO biology process (A-C) and KEGG pathway enrichments (D) were performed by mapping the host genes of dysregulated circRNAs using DAVID online tool. *P* < 0.05 was used as the threshold to select significant GO terms and KEGG pathways. -lg (*P* value) was the negative log10 of *P* value. Gene oncology (GO) enrichment analysis was made up of molecular function (A), biological process (B), and cellular component (C). The top 5 GO biology processes according to -lg (*P* value) and the Top 5 KEGG pathways were annotated. GO, Gene Ontology; BP, biology process; KEGG, Kyoto Encyclopedia of Genes and Genomes.

circRNAs are usually generated from the exons or introns of host genes and can regulate the expression of host genes 23. Hence, it is feasible to predict the function of circRNAs through their host genes. GO enrichment analysis showed that the most significant enriched GO term in molecular function was “Enzyme binding” (Figure 2A). The most significant enriched GO term in Biologic process was “Nervous system development” (Figure 2B). The most significant enriched GO term in cellular component was “Plasma membrane part” (Figure 2C). We also performed KEGG pathway analysis to determine the signaling pathways in which the host genes of differentially expressed circRNAs were involved. KEGG pathway analysis revealed the Top 5 enriched signaling pathways involved in the progression of MCAO, including pathways in cancer, lysosome, long-term potentiation, phosphatidylinositol signaling system, and glucagon signaling pathway. Of them, pathways in cancer signaling pathway was ranked the Top 1 (Figure 2D).

### cGLIS3 silencing alleviates MCAO-induced cerebral injury

**Figure 3:**
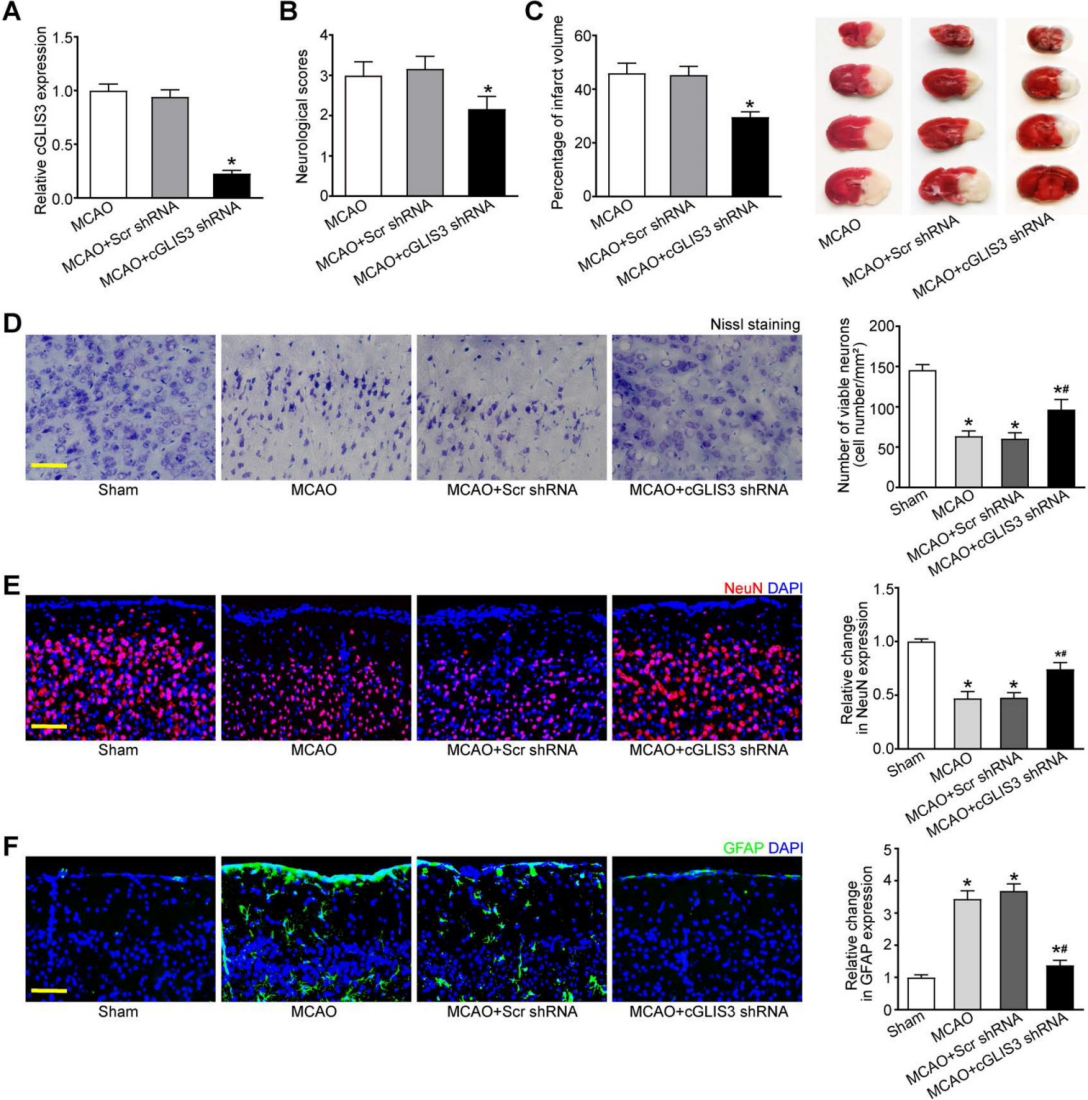
** GLIS3 silencing alleviates MCAO-induced cerebral injury.** (A) Stereotactic injection of scrambled (Scr) shRNA or cGLIS3 shRNA in cerebral cortex was performed before MCAO surgery. MCAO was performed to induce cerebral I/R injury. qRT-PCRs were performed to detect the levels of cGLIS3 expression (n = 6). (B) Bederson scoring was performed to determine the behavioral function at day 1 after MCAO (n = 6). (C) TTC staining assays were used to detect the size of ischemic cerebral infarction (n = 6). Representative images of 4 coronal sections stained with TTC at day 1 after MCAO. (D) Nissl's staining was performed on the coronal section of cerebral tissues to detect neuronal apoptosis at day 1 after MCAO (n = 6). Scale bar: 50 µm. (E) Immunofluorescence and quantitative analysis of NeuN staining was performed to detect the neuronal population. The representative images were shown (n = 6). Nuclei, blue; NeuN-positive cells, red. Scale bar: 50 µm. (F) Immunofluorescence and quantitative analysis of GFAP staining was performed to detect reactive astrocytes. The representative images were shown (n = 6). Nuclei, blue; GFAP-positive cells, green. Scale bar: 50 µm. *P < 0.05 versus Sham group; ^#^*P* < 0.05 MCAO+Scr shRNA group versus MCAO + cGLIS3 shRNA group. The significant difference was evaluated by the Kruskal-Wallis test followed by the post hoc Bonferroni test.

MCAO model is a widely used animal model for cerebral ischemia research. Since cGLIS3 was verified to be the most up-regulated circRNA in MCAO group, we thus investigated the role of cGLIS3 in ischemia-induced cerebral injury in the following study. The mice were subjected to MCAO to induce focal cerebral ischemia. They were killed at 6 hours, 24 hours, 3 days, 1 week, and 2 weeks after the onset of MCAO. qRT-PCR assays showed the levels of cGLIS3 were significantly up-regulated compared with that in the sham-operation group, suggesting that the induction of cGLIS3 could persist up to at least 2 weeks (Figure S1). The mice were injected with cGLIS3 shRNA or Scr shRNA two week before MCAO. qRT-PCR assays showed injection of cGLIS3 shRNA led to a marked reduction of cGLIS3 expression (Figure 3A). Neurological severity scores (NSS) tests showed that the score of MCAO mice after cGLIS3 silencing was significantly lower than that of MCAO+Scr shRNA group or MCAO group, suggesting that MCAO-raised score was reduced by the injection of cGLIS3 shRNA but not Scr shRNA (Figure 3B). TTC staining was performed to evaluate the degree of ischemic cerebral injury. Normal cerebral tissue was ruddy, while cerebral infarct area was pale. cGLIS3 shRNA injection reduced the size of cerebral infarct area (Figure 3C). Nissl staining was performed on the coronal section of cerebral tissue to evaluate the apoptosis of cortical neurons. There was a multiple of neuronal necrosis and irregularly arranged Nissl substances in MCAO group and Scr shRNA group. By contrast, the number of Nissl-positive neurons was significantly increased in cGLIS3 shRNA group (Figure 3D). NeuN staining was performed to detect the neuronal function in cerebral cortex. cGLIS3 silencing led to increased NeuN signaling in the cerebral cortex (Figure 3E). The degree of astrocytosis was determined by GFAP staining. cGLIS3 silencing led to decreased GFAP staining signaling in the cerebral cortex (Figure 3F). We also determined whether the change of baseline cGLIS3 level could affect cerebral injury* in vivo*. The results showed that cGLIS3 silencing did no further alter neurological severity score, cerebral infarct size, neuronal apoptosis, NeuN signaling, and GFAP signaling in sham-operation group (Figure S2).

### cGLIS3 silencing alleviates ischemia-induced retinal neurodegeneration

**Figure 4:**
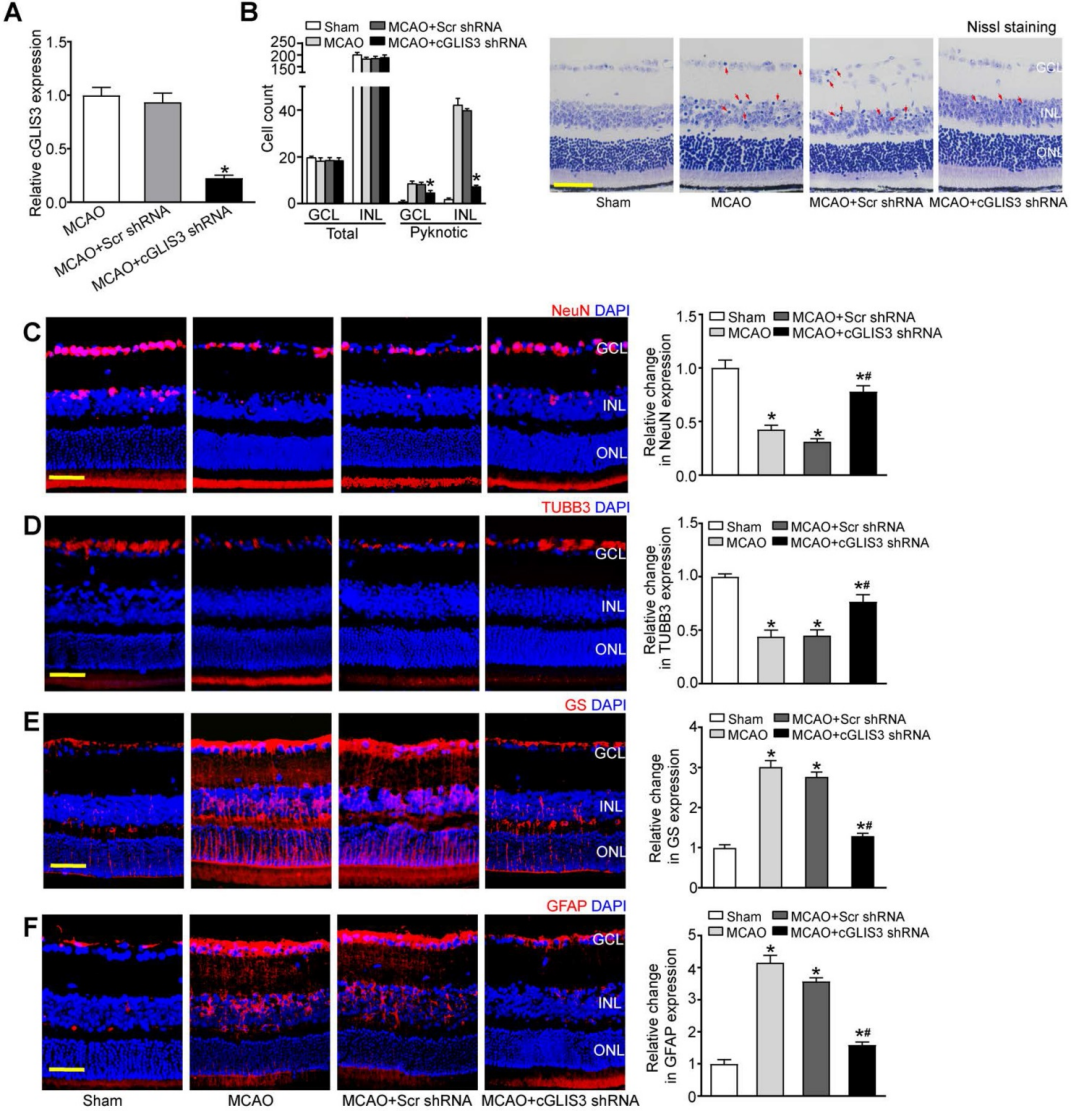
** cGLIS3 silencing alleviates ischemia-induced retinal neurodegeneration.** (A) The mice received an intravitreal injection of adeno-associated virus (AAV) containing cGLIS3 shRNA or scrambled (Scr) shRNA at 2 weeks before MCAO surgery. qRT-PCRs were performed to detect retinal cGLIS3 expression (n = 6) (B) The eyes were oriented and serially sectioned. Nissl staining and quantitative analysis were performed to detect the condensed pyknotic nuclei at day 1 after MCAO. The representative images and quantitative results were shown (n = 6). Scale bar: 50 µm. (C-F) Immunofluorescence staining assays with NeuN, TUBB3, GS, and GFAP were performed to determine RGC survival and reactive gliosis. The representative images and quantitative analysis were shown (n = 6). Nuclei, blue; NeuN-positive cells, red; TUBB3-positive cells, red; GS-positive cells, red; GFAP-positive cells, red. Scale bar: 50 µm. *P < 0.05 versus Sham group; ^#^*P* < 0.05 MCAO + Scr shRNA group versus MCAO + cGLIS3 shRNA group. The significant difference was evaluated by the Kruskal-Wallis test followed by the post hoc Bonferroni test.

MCAO also induces the occlusion of ophthalmic artery that supplies the retina. We then investigated whether cGLIS3 silencing could affect retinal neurodegeneration *in vivo*. Injection of cGLIS3 shRNA led to reduced cGLIS3 expression in the retinas (Figure 4A). Nissl staining showed that cGLIS3 silencing decreased the number of pyknotic nuclei compared with Scr shRNA-injected group or MCAO group (Figure 4B). MCAO can lead to RGC loss and astrocyte activation. Compared with the scrambled shRNA-injected retina, cGLIS3 shRNA injection alleviated MCAO-induced RGC injury as shown by increased NeuN staining and increased TUBB3 staining (Figure 4C-D), and inhibited retinal reactive gliosis as shown by decreased GS staining and GFAP staining (Figure 4E-F). Collectively, these results suggest that cGLIS3 silencing plays a protective role in MCAO-induced retinal neurodegeneration *in vivo*. We also determined whether the change of baseline cGLIS3 level affected retinal neurodegeneration *in vivo*. The results showed that cGLIS3 silencing did not affect retinal cell apoptosis, RGC survival, and astrocyte activation in sham-operation group (Figure S3).

### cGLIS3 silencing protects against OGD/R-induced neuronal cell injury

**Figure 5:**
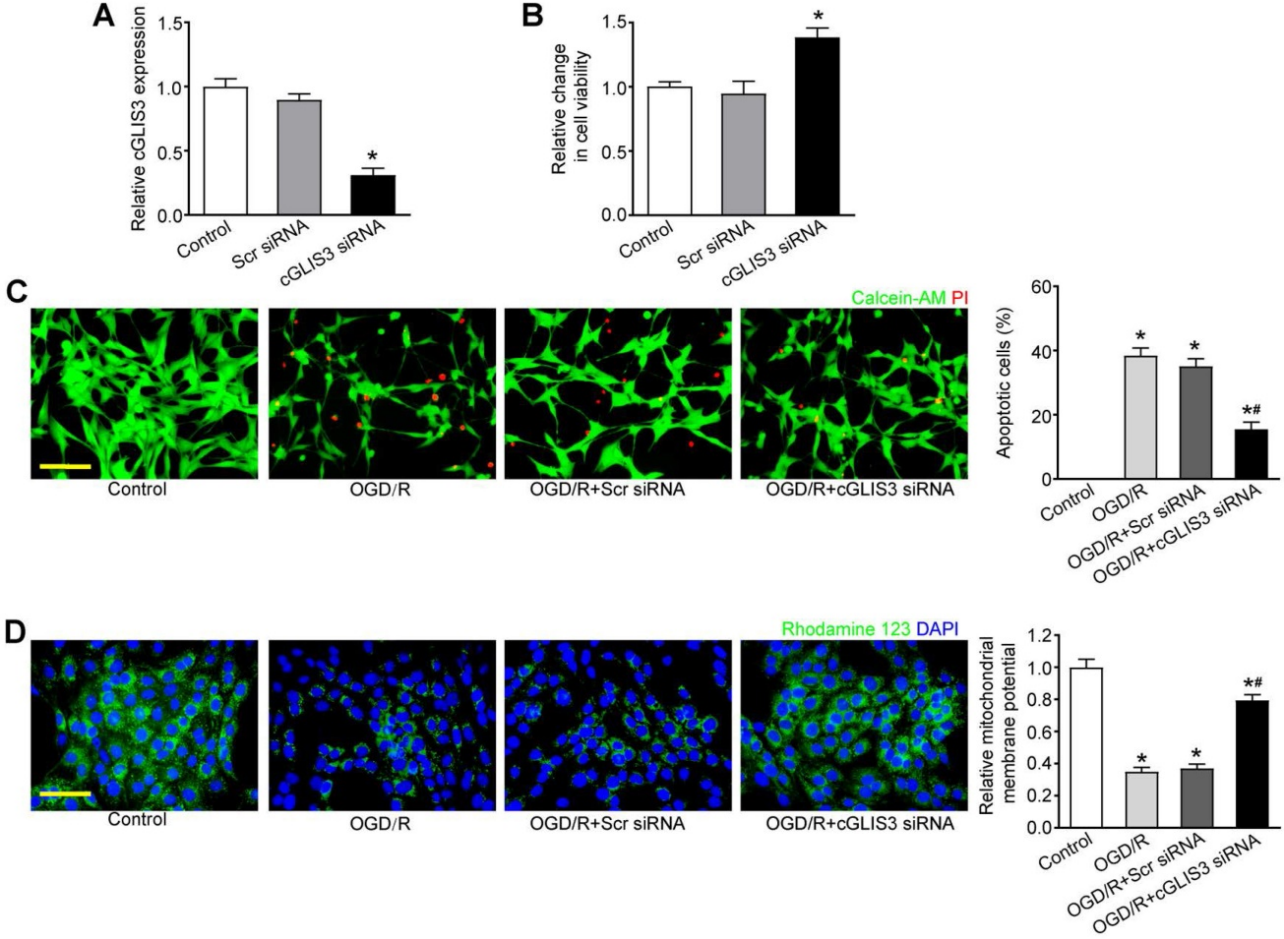
** cGLIS3 regulates RGC function *in vitro.***(A) RGCs were transfected with scrambled (Scr) siRNA, cGLIS3 siRNA, or left untreated (Ctrl). qRT-PCRs were performed to detect cGLIS3 expression levels (n = 3). (B-D) After transfection, RGCs were exposed to OGD for 2 h and reoxygenated for 24 h. MTT assays were performed to detect RGC viability (B, n = 3). The live and apoptotic RGCs were determined using Calcein-AM/PI double staining (C, n = 3). Calcein-positive cells, green; PI-positive cells, red. The number of apoptotic cells was counted by image-Pro Plus 6.0, Scale bar: 50 µm. Rhodamine staining was performed to detect the mitochondrial membrane potential (D, n = 3). Representative images with quantification results were shown. Nuclei, blue; Rhodamine 123, green. Scale bar, 50 µm. *P < 0.05 versus Control group; ^#^*P* < 0.05 OGD/R + cGLIS3 siRNA versus OGD/R + Scr siRNA. The significant difference was determined by one-way ANOVA followed by post-hoc Bonferroni's comparison test.

We further investigated the role of cGLIS3 in ischemia-induced retinal neuronal apoptosis *in vitro*. qRT-PCR assays showed that transfection of cGLIS3 siRNA led to decreased cGLIS3 expression in RGCs (Figure 5A). MTT assays showed that cGLIS3 silencing alleviated OGD/R-induced viability reduction of RGCs (Figure 5B). Calcein-AM/Propidium Iodide (PI) double staining showed that cGLIS3 silencing reduced OGD/R-induced apoptosis in RGCs (Figure 5C). Rhodamine 123 assays showed that cGLIS3 silencing further decreased OGD/R-induced reduction of ΔΨm in RGCs (Figure 5D). By contrast, cGLIS3 silencing did not affect RGC apoptosis under normal condition (Figure S4).

### cGLIS3 is a potential biomarker in ischemia-induced neurodegenerative disease

**Figure 6:**
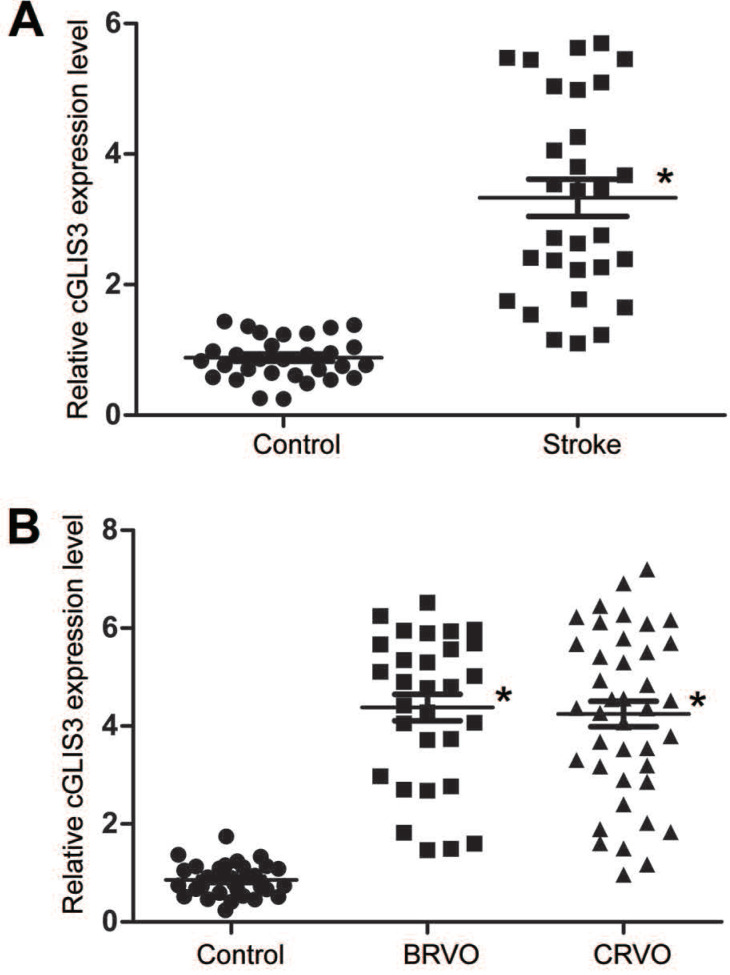
** cGLIS3 is a potential biomarker in ischemia-induced neurodegenerative disease.** (A) The serum samples were collected from the patients with ischemic stroke (n = 30) and healthy control (n = 30). qRT-PCR assays were performed to detect the levels of cGLIS3. The significant difference was evaluated by Student's *t*-test followed by the post hoc Bonferroni's test. (B) The AH samples were collected from the patients with CRVO (n = 40), BRVO (n = 30), and cataract (Control, n = 30). qRT-PCR assays were performed to detect the levels of cGLIS3. The significant difference was determined by one-way ANOVA followed by post-hoc Bonferroni's comparison test.

We then determined whether cGLIS3 up-regulation had the diagnostic value in ischemia-induced neurodegenerative diseases. 30 patients with ischemic stroke (62 ± 7.83 years old) and 30 healthy control subjects (59 ± 9.85 years old) were recruited. The level of circulating cGLIS3 was significantly increased in the patients with ischemic stroke compared to the healthy controls (Figure 6A). The levels of cGLIS3 in the patients with CRVO or BRVO were significantly higher than the control group (Figure 6B).

### cGLIS3 regulates OGD/R-induced injury by acting as miR-203 sponge *in vitro*

**Figure 7:**
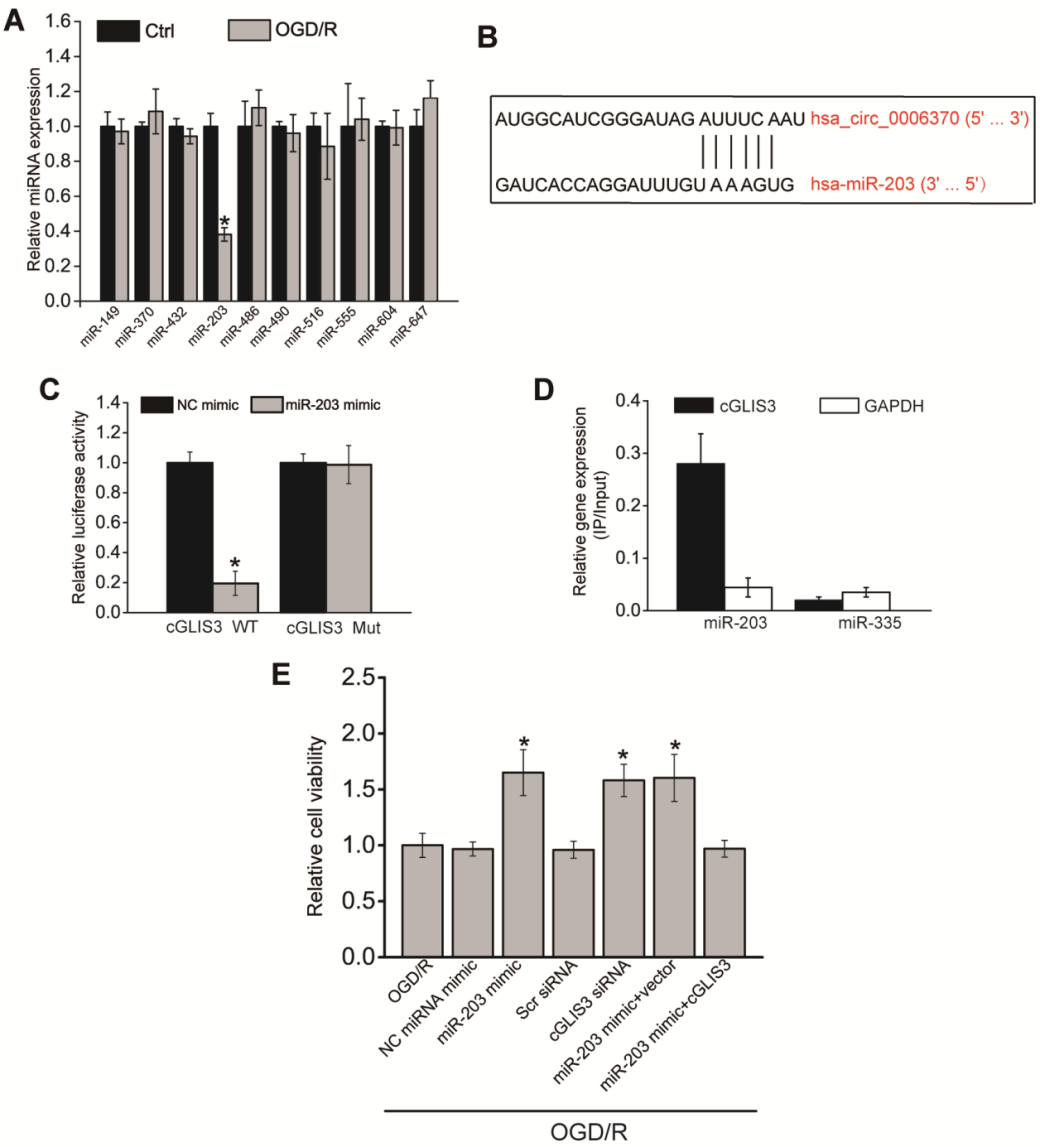
** cGLIS3 regulates OGD/R-induced injury by acting as miR-203 sponge *in vitro.***(A) RGCs were cultured in DMEM medium with deoxygenated glucose-free Hanks' Balanced Salt Solution for 1 h, transferred to hypoxic chamber with 0.2% O_2_ for 2 h, and then maintained under normoxic conditions for 24 h. As the control group, RGCs were grown in the media containing glucose under normal conditions. qRT-PCRs were performed to detect the levels of miRNAs (n = 4, *P < 0.05). (B) The binding sites on cGLIS3 for miR-203 were shown. (C) The entire cGLIS3 sequence was cloned into the pGL3 Luciferase Reporter to construct LUC-cGLIS3 wild-type vector (WT). Mutation of miR-203-binding sites in cGLIS3 sequence was performed to construct LUC-cGLIS3 mutant (Mut) vector. HEK293T cells were co-transfected LUC-cGLIS3 mutant or LUC-cGLIS3 WT with miR-203 mimic or control mimic. Luciferase activity assay was performed using the dual luciferase assay at 24 h after transfection (n = 4, * *P*< 0.05). (D) The 3ʹ-end biotinylated miR-203 or miR-335 (negative control group) duplexes were transfected into RGCs. After the streptavidin capture, the levels of cGLIS3 and GAPDH mRNA (negative control group) in the input and bound fractions were detected by qRT-PCR assays. Relative immunoprecipitate (IP)/input ratios were plotted. (E) RGCs were transfected as shown. They were cultured in DMEM medium with deoxygenated glucose-free Hanks' Balanced Salt Solution for 1 h, transferred to hypoxic chamber with 0.2% O_2_ for 2 h, and then maintained under normoxic conditions for 24 h. MTT assays were performed to detect cell viability (n = 4, *P < 0.05). The significant difference was determined by one-way ANOVA followed by post-hoc Bonferroni's comparison test.

Previous studies have revealed that circRNAs can regulate gene expressions by acting as miRNA sponge 24, 25. We speculated that cGLIS3 also regulated ischemia-induced neurodegeneration by acting as miRNA sponge. We searched Circular RNA Interactome (https://circinteractome.nia.nih.gov/) to identify the potential miRNAs interacting with cGLIS3. qPCR assays showed that only miR-203 was down-regulated in RGCs after OGD/R-induced injury (Figure 7A), indicating the association of miR-203 with OGD/R injury. We thus selected miR-203 for further investigation. The binding sites on cGLIS3 for miR-203 were shown (Figure 7B). Luciferase reporter assays were performed to investigate the interaction between miR-203 and cGLIS3. Overexpression of miR-203 led to decreased luciferase activity of wild-type (WT)-cGLIS3, instead of mutant (mut)-cGLIS3 (Figure 7C). Furthermore, using a biotin-coupled miR-203, we observed the greater enrichment of cGLIS3 in miR-203-captured fraction compared with the control group, biotinylated miR-335 (Figure 7D). We then determined the role of miR-203 in OGD/R-induced RGC injury *in vitro*. MTT assays showed that transfection of miR-203 mimic could alleviate OGD-induced reduction of cell viability, which could mimic the effect of cGLIS3 silencing on cell viability. cGLIS3 overexpression abrogated the protective effect of miR-203 on RGC viability (Figure 7E).

### EIF4A3 induces cGLIS3 expression *in vitro*

**Figure 8:**
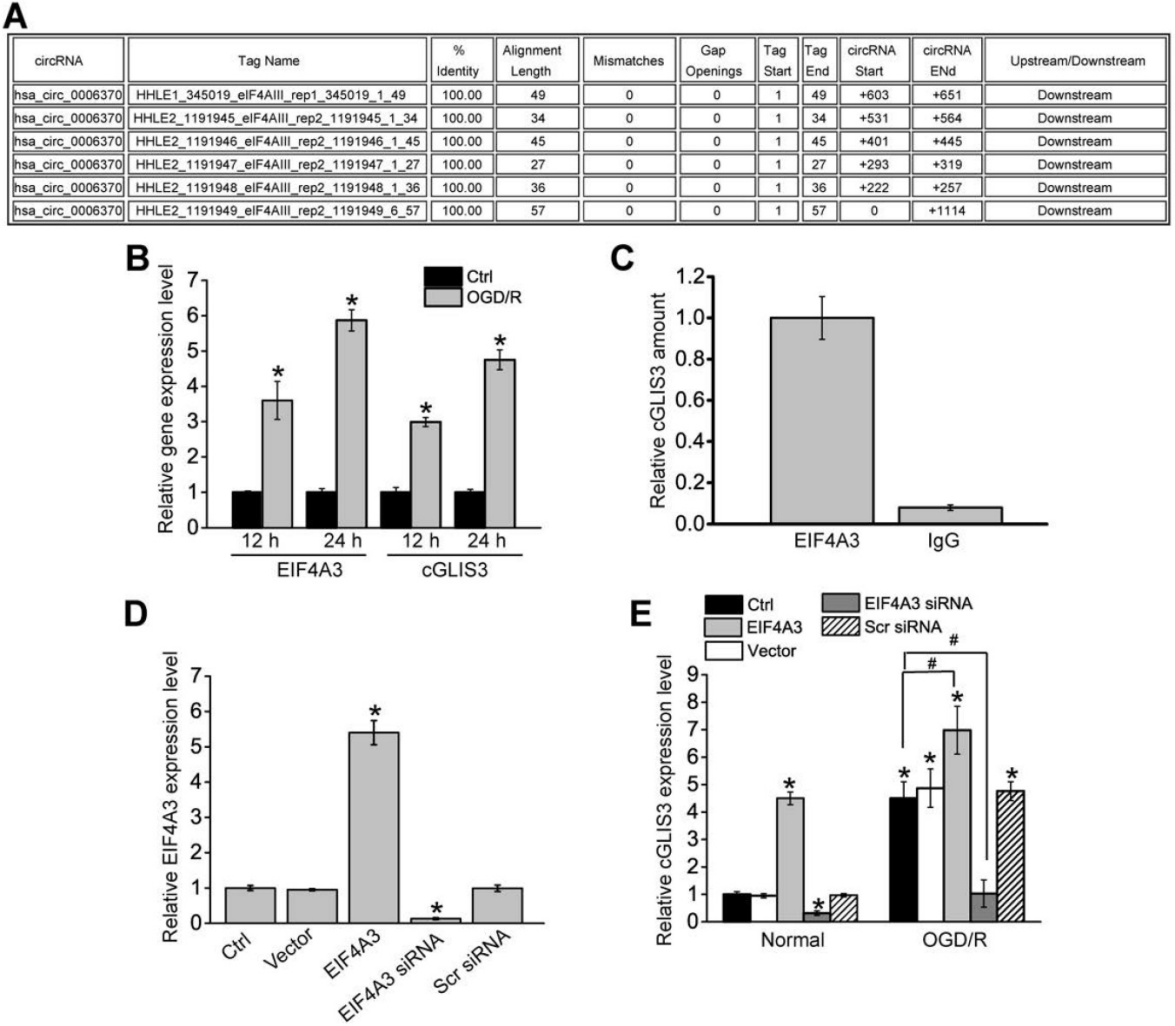
** EIF4A3 induces cGLIS3 expression in RGCs.** (A) The binding sites for EIF4A3 on GLIS3 transcript at the upstream region of cGLIS3 were obtained from Circular RNA Interactome. (B) RGCs were cultured in DMEM medium with deoxygenated glucose-free Hanks' Balanced Salt Solution for 1 h, transferred to hypoxic chamber with 0.2% O_2_ for 2 h. and then maintained under normoxic conditions for 12 h or 24 h. As the control group, normal cells were grown in the media containing glucose under normal conditions. qRT-PCRs were performed to detect the levels of EIF4A3 and cGLIS3. (C) Total cellular fractions were isolated from RGCs and immunoprecipitated using EIF4A3 or IgG antibody. The amount of cGLIS3 in the immunoprecipitate was detected by qRT-PCR assay (n = 4, *P < 0.05). (D) RGCs were transfected with pcDNA3.0-EIF4A3, pcDNA3.0 (Vector), EIF4A3 siRNA, scrambled (Scr) siRNA, or left untreated for 12 h. qRT-PCRs were performed to detect EIF4A3 levels (n = 4, *P < 0.05). (E) RGCs were treated as shown, and cultured in DMEM medium with deoxygenated glucose-free Hanks' Balanced Salt Solution for 1 h, transferred to hypoxic chamber with 0.2% O_2_ for 2 h. and then maintained under normoxic conditions for 12 h. qRT-PCRs were performed to detect the levels of cGLIS3 in the normal group and OGD/R group (n = 4, *P < 0.05). The significant difference was determined by one-way ANOVA followed by post-hoc Bonferroni's comparison test.

We further investigated the upstream regulatory mechanism of cGLIS3 induction. We retrieved the Circular RNA Interactome (https://circinteractome.nia.nih.gov/) and found that EIF4A3 had 6 binding sites on the upstream region of cGLIS3 transcript (Figure 8A). Eukaryotic initiation factor 4A-3 (EIF4A3) is a core component of the exon junction complex (EJC). eIF4A3 can bind to the host gene mRNA transcript, induce circRNA cyclization and increase circRNA expression 26,27. OGD/R treatment led to increased levels of EIF4A3 compared with the control group, which was consistent with increased levels of cGLIS3 (Figure 8B). To determine whether EIF4A3 can bind to cGLIS3, we performed RIP assays using EIF4A3 antibody, followed by qRT-PCR assays. cGLIS3 was enriched in EIF4A3-IP but not in Ago2-IP (Figure 8C). We also investigated whether overexpression and knockdown of EIF4A3 could alter cGLIS3 expression. qRT-PCR assays showed that overexpression of EIF4A3 led to increased levels of EIF4A3. By contrast, knockdown of EIF4A3 led to reduced levels of EIF4A3 (Figure 8D). Overexpression of EIF4A3 led to induced cGLIS3 expression both at the normal condition and OGD/R condition. By contrast, knockdown of EIF4A3 led to reduced cGLIS3 expression (Figure 8E). Collectively, the above-mentioned data suggests that EIF4A3 acts as an upstream regulator of cGLIS3.

## Discussion

Ischemia is a common pathological factor in many neurodegenerative diseases. Ischemia-induced neurodegeneration is a complicated pathological process, which is regulated by complex gene regulatory networks 28. However, the detailed mechanism remains to be clarified. circRNAs have shown as the versatile players in many biological processes and disease progression 14. Herein, we built an intraluminal middle cerebral artery occlusion (MCAO) model and identified 217 differentially expressed circRNAs between MCAO group and Sham group. MCAO also results in the occlusion of ophthalmic artery that supplies the retina 29. Among these differentially expressed circRNAs, silencing of cGLIS3 could alleviate MCAO-induced cerebral injury and ischemia-induced retinal neurodegeneration. This study provides a theoretical basis for further exploration of circRNA-mediated mechanism in neurodegeneration.

Ischemia is usually caused by the inadequacy of blood flow into a tissue. It can destroy the homeostatic balance and lead to neuronal cell dysfunction 30. In the central nervous system, ischemic injury can evoke neuronal death and glial activation 31. We showed that cGLIS3 was significantly up-regulated in ischemia-induced neurodegenerative models. cGLIS3 silencing decreased MCAO-induced apoptosis of cerebral neurons, reduced cerebral infarct size, and reduced astrocyte activation. cGLIS3 silencing also alleviated MCAO-induced retinal neuronal apoptosis, decreased RGC loss, and inhibited glial activation during retinal neurodegeneration. Thus, cGLIS3 silencing plays a protective role in ischemia-induced neurodegeneration.

The retina is formed as an outpouching of the diencephalon, which is known as the extension of cerebral tissue 32. It displays many similarities to cerebral tissue in terms of anatomy, functionality, stress response, and immunology 33. Several neurodegenerative changes in cerebral tissues have similar manifestations in the retina. The apoptotic pathways initiated upon ischemic damage in retina reflect the signaling found in other areas of central nervous system undergoing the similar injury 34, 35. Moreover, increasing evidence has shown that the patients with stroke often have visual dysfunction, which is associated with optic nerve damage, loss of ganglion cells, glia activation, and retinal thinning 36. Herein, we show that MCAO-induced cerebral ischemia injury and ischemia-induced retinal neurodegeneration shares the common regulator and similar regulatory mechanism. The retina is also more accessible than other areas of central nervous system, thus making it a simpler model for study. By utilizing retinal model, we can greatly increase our knowledge about neurodegenerative processes initiated by ischemia which lead to degeneration in the central nervous system.

Due to high stability, specific expression pattern, and wide distribution in body fluids, circRNAs have shown as the promising biomarkers for many human diseases 37. Stroke is one of the leading causes of death and disability. When stroke occurs, oxygen and glucose perfusion are restricted in a specific cerebral region, leading to neural cell death 38. Clinical evidence shows that cerebral injury often occurs in the hours to days after stroke. Only a small time-frame is left for therapeutic intervention 39. Although several biomarkers have been assessed, none have been proven to be reliable enough in the clinical settings. Hence, it is still required to develop a new method for the early diagnosis of stroke, such as the use of blood biomarker. The level of circulating cGLIS3 is significantly greater in the patients with ischemic stroke compared to healthy controls, suggesting that the potential utility of cGLIS3 as a blood biomarker to be used singly or as part of a biomarker panel in the patients with stroke.

The translation of a blood-based biomarker in ischemic stroke to the clinical practice is challenging due to disease complexity and the presence of blood-brain barrier that restricts the release of brain-specific markers into the circulation. To further verify the diagnostic value of cGLIS3, we also collected aqueous humor (AH) from the patients with retinal vein occlusion (RVO). Retinal vein occlusion (RVO) is usually classified into branch RVO (BRVO) and central RVO (CRVO) according to the anatomical site of vascular occlusion 40. The pathogenesis of RVO is associated with the blockage of ocular circulation, leading to elevated intraluminal pressure and subsequent hemorrhages and leakage of fluid within the retina 41. The pathogenesis of RVO is also tightly associated with retinal ischemia. AH is a dynamic intraocular fluid that regulates intraocular pressure 42. The circulation of AH occurs in a highly relative closed system. Its condition is tightly associated with ocular diseases 43. The levels of cGLIS3 in AH of RVO patients were significantly higher than that in the control group. Increased cGLIS3 level may affect retinal neuronal function and retinal neurodegenerative process during RVO. Therefore, monitoring cGLIS3 levels could be used for the diagnosis of ischemia-induced neurodegenerative diseases.

## Conclusions

We provided a transcriptome-wide overview of aberrantly expressed circRNAs in neurodegeneration and identified cGLIS3 as a novel biomarker for ischemia-induced neurodegenerative diseases. We also preliminarily investigated the role of cGLIS3 in neurodegeneration. In future study, we will investigate the functional significance and regulatory mechanism of identified circRNAs in neurodegeneration and provided novel insights into the treatment of neurodegenerative diseases.

## Supplementary Material

Supplementary figures and tables.

## Figures and Tables

**Table 1:** Verification of microarray data by qRT-PCRs

circRNA name	Ischemia versus Control			
	Microarray		qRT-PCR	
cGlis3	22.83	up	16.47	up
cZranb1	15.40	up	4.93	up
cCyb5r4	10.82	up	6.84	up
cZfp609	10.49	up	3.58	up
cPtprn2	10.48	up	12.43	up
cNyap2	8.07	up	1.98	up
cDab1	7.49	up	3.28	up
cErc2	7.49	up	1.14	Not verified
cDlx6os1	7.00	up	5.85	up
cPtprr	6.97	up	3.04	up
cDpy19l1	0.22	down	0.28	down
cDennd2c	0.21	down	1.23	Not verified
cGfra1	0.21	down	0.17	down
cAp1s2	0.21	down	0.97	Not verified
cAurkb	0.21	down	0.09	down
cRsf1	0.20	down	0.14	down
cCacna1e	0.17	down	0.37	down
cCse1l	0.14	down	0.13	down
cKlhl14	0.11	down	0.25	down
cUqcc1	0.08	down	0.06	down

## References

[1] Tymianski M (2011). Emerging mechanisms of disrupted cellular signaling in brain ischemia. Nat Neurosci.

[2] Caprara C, Grimm C (2012). From oxygen to erythropoietin: relevance of hypoxia for retinal development, health and disease. Prog Retin Eye Res.

[3] Bargiotas P, Krenz A, Hormuzdi SG, Ridder DA, Herb A, Barakat W (2011). Pannexins in ischemia-induced neurodegeneration. Proc Natl Acad Sci U S A.

[4] Attwell D, Buchan AM, Charpak S, Lauritzen M, Macvicar BA, Newman EA (2010). Glial and neuronal control of brain blood flow. Nature.

[5] Eltzschig HK, Eckle T (2011). Ischemia and reperfusion-from mechanism to translation. Nat Med.

[6] Carbonell T, Gomes AV (2020). MicroRNAs in the regulation of cellular redox status and its implications in myocardial ischemia-reperfusion injury. Redox Biol.

[7] Veenith TV, Carter EL, Geeraerts T, Grossac J, Newcombe VF, Outtrim J (2016). Pathophysiologic mechanisms of cerebral ischemia and diffusion hypoxia in traumatic brain injury. JAMA Neurol.

[8] Kristensen LS, Andersen MS, Stagsted LVW, Ebbesen KK, Hansen TB, Kjems J (2019). The biogenesis, biology and characterization of circular RNAs. Nat Rev Genet.

[9] Zlotorynski E (2019). The innate function of circular RNAs. Nat Rev Mol Cell Biol.

[10] Nicolet BP, Engels S, Aglialoro F, van den Akker E, von Lindern M, Wolkers MC (2018). Circular RNA expression in human hematopoietic cells is widespread and cell-type specific. Nucleic Acids Res.

[11] Li X, Yang L, Chen LL (2018). The biogenesis, functions, and challenges of circular RNAs. Mol Cell.

[12] Han B, Chao J, Yao H (2018). Circular RNA and its mechanisms in disease: from the bench to the clinic. J Clin Pharm Ther.

[13] Cucchiara B, Elm J, Easton JD, Coutts SB, Willey JZ, Biros MH (2020). Disability after minor stroke and transient ischemic attack in the POINT trial. Stroke.

[14] Stinear CM, Lang CE, Zeiler S, Byblow WD (2020). Advances and challenges in stroke rehabilitation. Lancet Neurol.

[15] Mulder MJHL, Jansen IGH, Goldhoorn RB, Venema E, Chalos V, Compagne KCJ (2018). Time to endovascular treatment and outcome in acute ischemic stroke: MR CLEAN registry results. Circulation.

[16] Cheung CY, Ikram MK, Chen C, Wong TY (2017). Imaging retina to study dementia and stroke. Prog Retin Eye Res.

[17] Stefansson E, Olafsdottir OB, Eliasdottir TS, Vehmeijer W, Einarsdottir AB, Bek T (2019). Retinal oximetry: metabolic imaging for diseases of the retina and brain. Prog Retin Eye Res.

[18] Eggenberger ER (2019). Retinal occlusion, ischemic stroke, and the brain-eye connection. Mayo Clin Proc.

[19] Bai Y, Zhang Y, Han B, Yang L, Chen XF, Huang RR (2018). Circular RNA DLGAP4 ameliorates ischemic stroke outcomes by targeting miR-143 to regulate endothelial-mesenchymal transition associated with blood-brain barrier integrity. J Neurosci.

[20] Wu FF, Han B, Wu SS, Yang L, Leng S, Li MY (2019). Circular RNA TLK1 aggravates neuronal injury and neurological deficits after ischemic stroke via miR-335-3p/TIPARP. J Neurosci.

[21] Wang JJ, Liu C, Shan K, Liu BH, Li XM, Zhang SJ (2018). Circular RNA-ZNF609 regulates retinal neurodegeneration by acting as miR-615 sponge. Theranostics.

[22] Wang JJ, Shan K, Liu BH, Liu C, Zhou RM, Li XM (2018). Targeting circular RNA-ZRANB1 for therapeutic intervention in retinal neurodegeneration. Cell Death Dis.

[23] Zhang SJ, Chen X, Li CP, Li XM, Liu C, Liu BH (2017). Identification and characterization of circular RNAs as a new class of putative biomarkers in diabetes retinopathy. Invest Ophthalmol Vis Sci.

[24] Shan K, Liu C, Liu BH, Chen X, Dong R, Liu X (2017). Circular noncoding RNA HIPK3 mediates retinal vascular dysfunction in diabetes mellitus. Circulation.

[25] Liu C, Yao MD, Li CP, Shan K, Yang H, Wang JJ (2017). Silencing of circular RNA-ZNF609 ameliorates vascular endothelial dysfunction. Theranostics.

[26] Wang R, Zhang S, Chen X, Li N, Li J, Jia R (2018). EIF4A3-induced circular RNA MMP9 (circMMP9) acts as a sponge of miR-124 and promotes glioblastoma multiforme cell tumorigenesis. Mol Cancer.

[27] Li Y, Ren S, Xia J, Wei Y, Xi Y (2020). EIF4A3-Induced circ-BNIP3 aggravated hypoxia-induced injury of H9c2 cells by targeting miR-27a-3p/BNIP3. Mol Ther Nucleic Acids.

[28] Kahl A, Blanco I, Jackman K, Baskar J, Milaganur Mohan H, Rodney-Sandy R (2018). Cerebral ischemia induces the aggregation of proteins linked to neurodegenerative diseases. Sci Rep.

[29] Ritzel RM, Pan SJ, Verma R, Wizeman J, Crapser J, Patel AR (2016). Early retinal inflammatory biomarkers in the middle cerebral artery occlusion model of ischemic stroke. Mol Vis.

[30] Yang Y, Sandhu HK, Zhi F, Hua F, Wu M, Xia Y (2015). Effects of hypoxia and ischemia on microRNAs in the brain. Curr Med Chem.

[31] Jaquins-Gerstl A, Shu Z, Zhang J, Liu Y, Weber SG, Michael AC (2011). Effect of dexamethasone on gliosis, ischemia, and dopamine extraction during microdialysis sampling in brain tissue. Anal Chem.

[32] London A, Benhar I, Schwartz M (2013). The retina as a window to the brain-from eye research to CNS disorders. Nat Rev Neurol.

[33] Muthaian R, Minhas G, Anand A (2012). Pathophysiology of stroke and stroke-induced retinal ischemia: emerging role of stem cells. J Cell Physiol.

[34] Koronyo-Hamaoui M, Koronyo Y, Ljubimov A, Miller C, Ko M, Black K (2011). Identification of amyloid plaques in retinas from Alzheimer's patients and noninvasive *in vivo* optical imaging of retinal plaques in a mouse model. NeuroImage.

[35] Dias-Santos A, Proença R, Tavares Ferreira J, Pinheiro S, Cunha J, Proença R (2018). The role of ophthalmic imaging in central nervous system degeneration in systemic lupus erythematosus. Autoimmun Rev.

[36] D'Onofrio P, Koeberle P (2013). What can we learn about stroke from retinal ischemia models?. Acta Pharmacol Sin.

[37] Zhang Z, Yang T, Xiao J (2018). Circular RNAs: promising biomarkers for human diseases. EBioMedicine.

[38] Bao MH, Szeto V, Yang BB, Zhu SZ, Sun HS, Feng ZP (2018). Long non-coding RNAs in ischemic stroke. Cell Death Dis.

[39] Shkirkova K, Saver JL, Starkman S, Wong G, Weng J, Hamilton S (2018). Frequency, predictors, and outcomes of prehospital and early postarrival neurological deterioration in acute stroke:exploratory analysis of the FAST-MAG randomized clinical trial. JAMA Neurol.

[40] Ponto KA, Scharrer I, Binder H, Korb C, Rosner AK, Ehlers TO (2019). Hypertension and multiple cardiovascular risk factors increase the risk for retinal vein occlusions: results from the Gutenberg Retinal Vein Occlusion Study. J Hypertens.

[41] Khayat M, Williams M, Lois N (2018). Ischemic retinal vein occlusion: characterizing the more severe spectrum of retinal vein occlusion. Surv Ophthalmol.

[42] Dismuke WM, Challa P, Navarro I, Stamer WD, Liu Y (2015). Human aqueous humor exosomes. Exp Eye Res.

[43] Friedenwald JS, Becker B (1956). Aqueous humor dynamics; theoretical considerations. AMA Arch Ophthalmol.

