# Supplementary material for: Retina as a window to cerebral dysfunction following studies with circRNA signature during neurodegeneration
Source: Theranostics. 2021 Jan 1;11(4):1814–27. doi: 10.7150/thno.51550 (PMC7778582; doi:10.7150/thno.51550)
Supplement: Supplementary file 1 — Supplementary figures and tables. [file thnov11p1814s1.pdf]

Supplemental Material

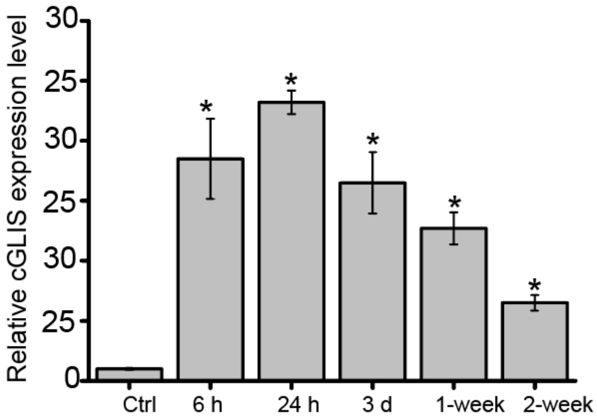

Figure S1: MCAO treatment leads to increased cGLIS3 expression

The mice were subjected to transient middle cerebral artery occlusion (tMCAO). They were killed at 6 hours, 24 hours, 3 days, 1-week, and 2-week after the onset of MCAO. qRT-PCR assays were conducted to detect the level of cGLIS3 expression (n = 6 animals per group). $*P < 0.05$ versus Ctrl group. The significant difference was evaluated by the Kruskal-Wallis test followed by the post hoc Bonferroni test.

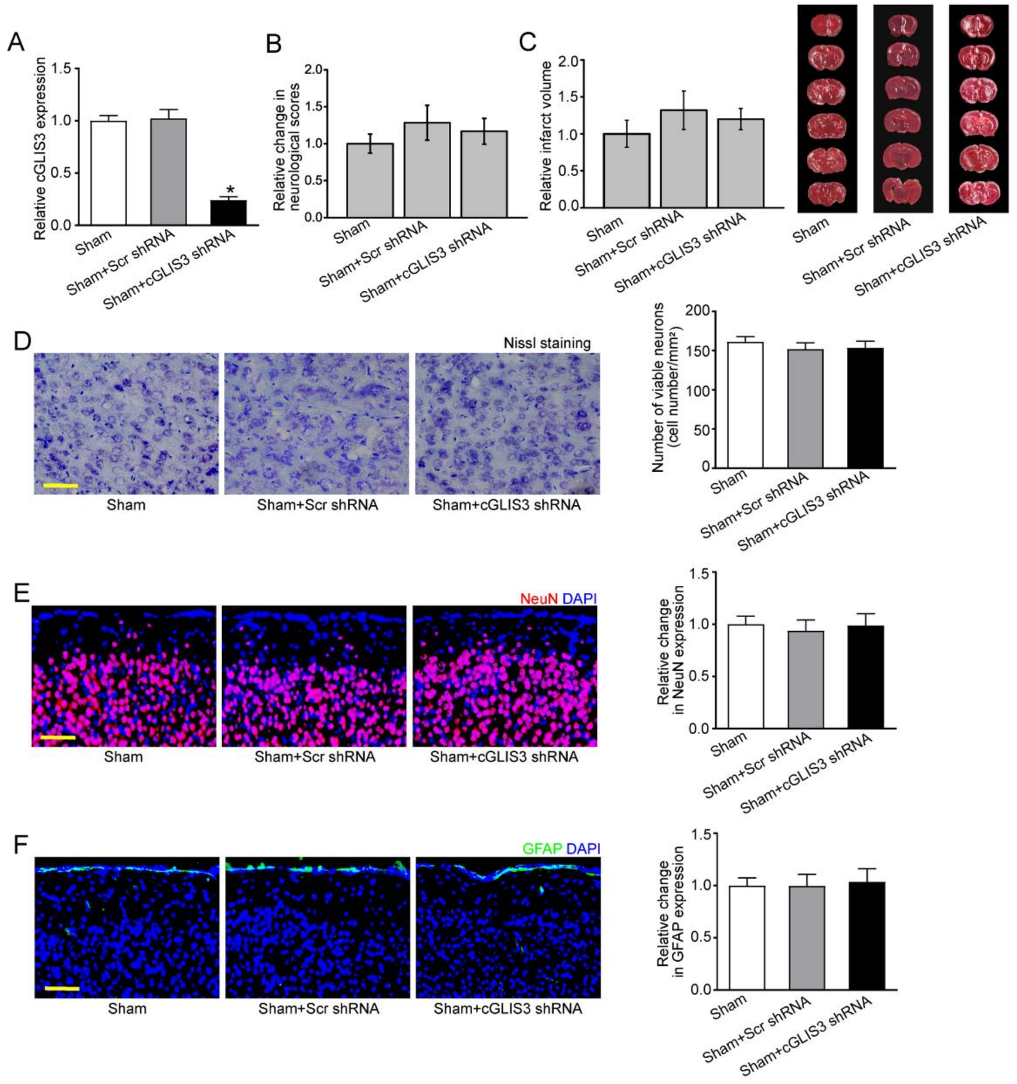

Figure S2: GLIS3 silencing has no effect on cerebral injury in Sham group

(A) The mice received the stereotactic injection of scrambled (Scr) shRNA or cGLIS3 shRNA in cerebral cortex at 2 weeks before Sham operation. qRT-PCRs were performed to detect the levels of cGLIS3 expression (n = 6). (B) Bederson scoring was performed to determine the behavioral function at day 1 after Sham operation (n = 6). (C) TTC staining assays were used to detect the size of ischemic cerebral infarction (n = 6). Representative images of 6 coronal sections stained with TTC at day 1 after Sham operation. (D) Nissl's staining was performed on the coronal section of cerebral tissue to detect cortical neuron apoptosis at day 1 after Sham operation (n = 6). Scale bar: 50 μm. (E) Immunofluorescence and quantitative analysis of NeuN staining was performed to detect neuronal population. The representative images were shown (n = 6). Nuclei, blue; NeuN-positive cells, red. Scale bar: 50 μm. (F) Immunofluorescence and quantitative analysis of GFAP staining was performed to detect reactive astrocytes. The representative images were shown (n = 6). Nuclei, blue; GFAP-positive cells, green. Scale bar: 50 μm. **P* < 0.05 versus Sham group. The significant difference was evaluated by the Kruskal-Wallis test followed by the post hoc Bonferroni test.

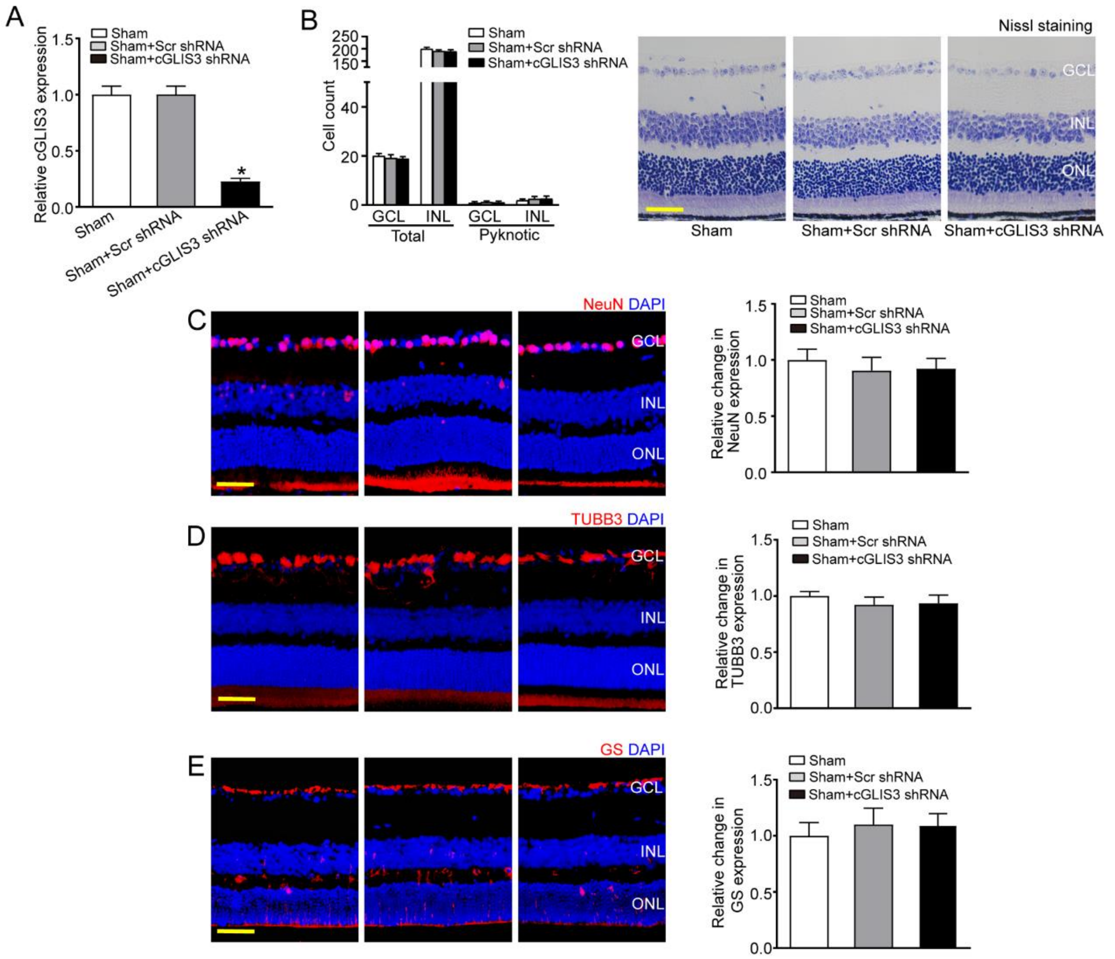

Figure S3: GLIS3 silencing has no effect on retinal neurodegeneration in Sham group

(A) The mice received an intravitreal injection of adeno-associated virus (AAV) containing cGLIS3 shRNA or scrambled (Scr) shRNA at 2 weeks before Sham operation. qRT-PCRs were performed to detect retinal cGLIS3 expression (n = 6) (B) Eyes were oriented and serially sectioned. Nissl staining and quantitative analysis was performed to detect the condensed pyknotic nuclei at day 1 after Sham operation. The representative images and quantitative results were shown (n = 6). Scale bar: 50 μm. (C-E) Immunofluorescence staining assays with NeuN, TUBB3, and GS were performed to determine RGC survival and reactive gliosis. The representative images and quantitative analysis were shown (n = 6). Nuclei, blue; NeuN-positive cells, red; TUBB3-positive cells, red; GS-positive cells, red. Scale bar: 50 μm. *$P < 0.05$ versus Sham group. The significant difference was evaluated by the Kruskal-Wallis test followed by the post hoc Bonferroni test.

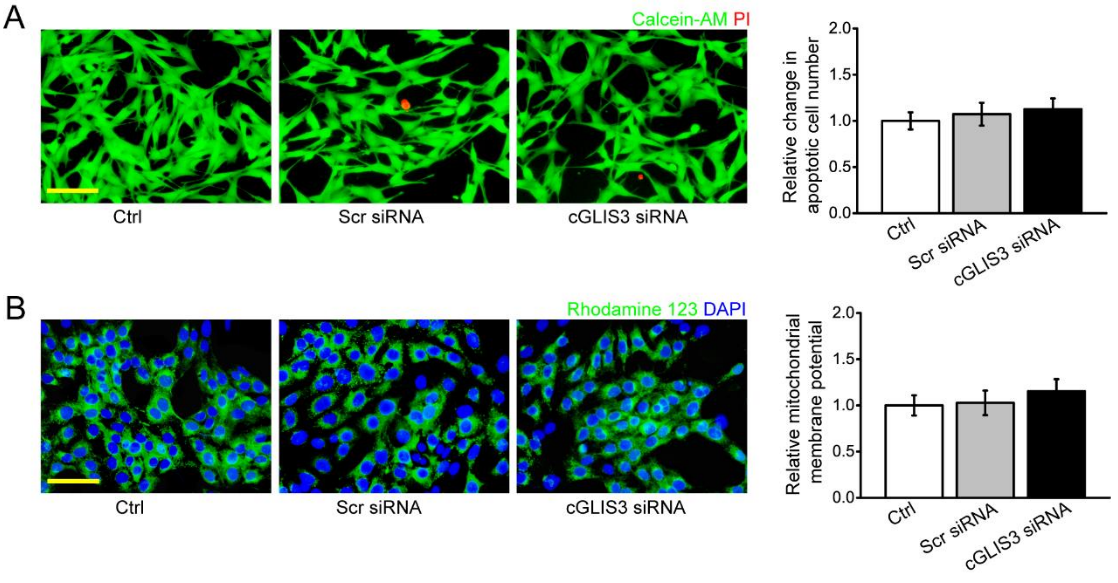

Figure S4: cGLIS3 has no effect on RGC apoptosis under normal condition

(A and B) RGCs were transfected with scrambled (Scr) siRNA, cGLIS3 siRNA, or left untreated (Ctrl) for 12 h. The live and apoptotic RGCs were determined using Calcein-AM/PI double staining (A, n = 3). Calcein-positive cells, green; PI-positive cells, red. The number of apoptotic cells was counted by Image-Pro Plus 6.0, Scale bar: 50 μm. Rhodamine staining was performed to detect the mitochondrial membrane potential (B, n = 3). Representative images with the quantification result were shown. Nuclei, blue; Rhodamine 123, green. Scale bar, 50 μm. The significant difference was determined by one-way ANOVA followed by post-hoc Bonferroni's comparison test.

Table S1: Differentially expressed circRNAs between MCAO group and sham-operation group

circRNA_ID	Sham 1	Sham 2	Sham 3	MCAO 1	MCAO 2	MCAO 3	Host gene	Fold change
mmu_circ_0000943	0.34	0.41	0.43	3.80	4.53	6.39	Glis3	22.83
mmu_circ_0013957	3.78	5.10	3.72	7.71	9.33	7.40	Zranb1	15.40
cicRNA.18946	1.39	1.80	2.17	6.20	4.55	4.92	Cyb5r4	10.82
mmu_circ_0001797	8.46	5.79	7.50	10.71	10.57	10.65	Zfp609	10.49
cicRNA.10465	0.33	0.57	0.74	2.33	5.08	4.39	Ptprn2	10.48
cicRNA.15752	8.02	8.93	7.13	12.61	10.07	10.43	Nyap2	8.07
cicRNA.23088	0.40	0.51	0.46	2.99	4.07	3.02	Dab1	7.49
mmu_circ_0005154	0.31	0.77	0.25	4.72	3.42	1.91	Erc2	7.49
cicRNA.20664	1.37	1.23	0.90	2.93	4.98	4.02	Dlx6os1	7.00
cicRNA.15205	0.41	0.54	0.51	2.43	3.55	3.89	Ptprr	6.97
mmu_circ_0007938	21.50	21.64	21.06	23.45	24.67	24.30	Malat1	6.69
cicRNA.13667	1.16	1.46	0.96	2.46	4.89	4.32	Anks1b	6.49
cicRNA.10463	0.39	0.34	0.35	2.83	2.76	3.53	Ptprn2	6.39
mmu_circ_0000767	19.49	20.15	19.67	21.95	22.40	22.84	None	6.18
cicRNA.10459	0.98	0.60	2.67	2.81	4.89	4.26	Ptprn2	5.95
cicRNA.22217	0.35	1.60	2.11	2.75	4.88	4.09	Pappa	5.88
mmu_circ_0008419	3.92	3.04	2.87	5.78	5.18	6.50	Olfml2b	5.84
cicRNA.22781	2.98	3.23	2.37	3.80	6.79	5.58	Sesn2	5.77
cicRNA.6620	0.79	0.58	0.55	1.75	3.45	4.21	Enox1	5.64
cicRNA.2737	20.39	20.79	20.27	22.36	23.12	23.28	Gm26917	5.42
mmu_circ_0006521	1.23	1.25	0.37	2.27	3.32	4.47	Dscam	5.29
cicRNA.20743	1.71	2.32	1.54	3.63	4.91	4.24	Ubn2	5.27
mmu_circ_0007958	19.88	20.00	19.75	22.11	22.52	22.20	Malat1	5.27
cicRNA.10651	0.58	0.57	0.58	1.65	4.44	2.83	n/a	5.27
cicRNA.2736	20.40	20.36	19.98	22.37	22.76	22.80	Gm26917	5.27
mmu_circ_0006756	19.25	19.89	19.25	21.56	21.30	22.58	None	5.10
cicRNA.27102	0.67	0.56	0.55	1.53	3.97	3.30	Myef2	5.06
cicRNA.2772	21.11	20.51	20.37	22.24	22.97	23.72	Gm26917	4.96
mmu_circ_0006757	19.31	19.92	19.48	21.63	21.47	22.54	None	4.95
cicRNA.26414	1.43	1.65	1.79	2.71	4.73	4.30	Rprd1b	4.90
mmu_circ_0001934	0.97	0.82	0.37	1.59	3.09	4.34	Syt1	4.87
cicRNA.8506	0.72	1.80	0.28	4.26	3.08	2.31	Fgd3	4.85
cicRNA.22473	1.84	1.86	0.78	2.96	4.32	3.89	Bach2	4.70
cicRNA.9304	1.02	0.56	1.53	3.03	2.91	3.84	Efcab11	4.67
cicRNA.16395	19.97	19.37	18.84	21.25	21.91	21.65	Map4k4	4.62
mmu_circ_0000783	18.64	18.95	18.44	20.28	21.00	21.27	None	4.52
cicRNA.23652	1.45	0.34	0.70	2.84	3.69	2.41	Trpc4	4.45
cicRNA.17157	19.98	19.71	19.74	21.45	22.35	22.07	Uap1	4.44
mmu_circ_0000834	2.64	0.45	0.52	3.07	3.36	3.61	None	4.41
cicRNA.2745	17.80	18.13	17.83	19.90	20.32	19.93	Gm26917	4.39

mmu_circ_0003918	0.32	0.30	0.29	2.31	3.17	1.79	Hdac9	4.36
cicRNA.15341	1.06	2.30	0.70	2.43	4.16	3.78	Ppfia2	4.29
mmu_circ_0000760	18.17	18.39	18.13	20.12	20.55	20.28	None	4.26
mmu_circ_0007957	24.05	23.88	23.43	25.94	25.43	26.25	Malat1	4.24
cicRNA.27736	19.01	19.25	18.81	21.16	20.92	21.24	Malat1	4.24
mmu_circ_0005281	17.25	17.74	17.40	19.40	19.66	19.57	RP24-90K1.5	4.24
mmu_circ_0000924	21.11	20.69	20.16	22.56	22.56	23.09	Malat1	4.23
cicRNA.10746	2.24	2.02	1.04	2.77	3.99	4.77	Unc79	4.22
mmu_circ_0011635	17.53	17.79	17.23	19.51	19.05	20.21	Igfbpl1	4.21
cicRNA.27741	21.16	20.57	20.18	22.58	22.50	23.05	Malat1	4.20
cicRNA.20351	18.32	18.06	18.04	19.76	20.56	20.30	n/a	4.19
cicRNA.7037	19.93	20.30	20.42	22.05	22.49	22.24	Rpph1	4.12
cicRNA.10652	1.21	0.34	0.52	3.24	3.40	1.55	n/a	4.12
mmu_circ_0000537	16.90	17.39	17.42	18.75	19.87	19.18	Rpph1	4.08
mmu_circ_0007941	17.68	17.59	16.97	19.14	19.68	19.50	Malat1	4.07
mmu_circ_0007944	20.28	20.40	20.13	22.34	22.11	22.43	Malat1	4.05
cicRNA.2773	19.05	18.64	18.53	20.23	20.72	21.31	Gm26917	4.03
cicRNA.9856	1.20	1.39	1.23	3.91	3.45	2.46	Mis18bp1	4.00
cicRNA.13683	0.29	0.31	0.31	1.20	2.74	2.97	Anks1b	4.00
cicRNA.5070	0.28	0.25	0.28	1.50	2.13	3.14	Sult4a1	3.97
cicRNA.2728	18.75	19.17	18.69	20.79	20.29	21.47	Gm26917	3.95
cicRNA.27734	21.85	21.54	21.48	24.01	22.60	24.19	Malat1	3.93
cicRNA.13804	1.79	0.64	1.90	2.92	3.05	4.30	Aldh1l2	3.93
cicRNA.6624	0.77	1.88	2.00	3.32	3.60	3.65	Enox1	3.92
cicRNA.179	16.64	16.40	16.12	17.82	19.11	18.10	Lrp5	3.88
cicRNA.6597	0.33	0.35	0.34	1.62	3.25	2.01	Enox1	3.88
mmu_circ_0002202	1.16	3.10	1.03	3.76	3.80	3.49	Armc2	3.79
cicRNA.1738	1.24	0.30	0.73	2.96	1.65	3.42	Nrxn1	3.78
cicRNA.6361	16.94	17.06	16.79	18.52	19.46	18.51	Gm28047	3.74
mmu_circ_0016383	0.48	0.84	0.56	2.27	2.35	2.94	Dmd	3.72
mmu_circ_0007935	17.48	17.64	17.42	19.43	18.91	19.88	Malat1	3.72
cicRNA.19607	0.28	0.31	0.32	1.11	3.34	2.10	Zcwpw2	3.68
mmu_circ_0003232	3.78	2.34	3.29	5.06	4.33	5.63	Arhgap44	3.65
mmu_circ_0007961	21.15	21.24	21.14	23.50	22.83	22.79	Malat1	3.64
cicRNA.27738	21.79	21.56	21.54	23.97	22.56	23.91	Malat1	3.60
cicRNA.20110	2.23	2.23	3.09	5.07	4.20	3.78	Dlgap2	3.57
mmu_circ_0007100	3.20	3.78	3.04	4.85	4.88	5.77	Nrxn1	3.55
cicRNA.3270	16.61	16.69	16.60	18.08	18.62	18.64	Qk	3.52
cicRNA.19856	2.67	2.59	3.26	4.61	4.41	4.89	Inpp4b	3.49
mmu_circ_0015020	1.15	0.79	1.24	2.27	2.88	3.43	Inpp4b	3.49
cicRNA.15116	17.06	17.42	16.89	18.99	18.66	19.11	Gns	3.48
cicRNA.12530	1.96	0.29	0.24	2.19	3.06	2.60	Tenm2	3.44

cicRNA.22624	1.96	0.29	0.24	2.19	3.06	2.60	Ddi2	3.44
cicRNA.23654	1.24	1.88	0.53	2.42	3.58	2.96	Trpc4	3.42
cicRNA.22161	0.31	0.33	0.34	0.92	2.58	2.79	Ccdc171	3.40
mmu_circ_0006279	0.94	1.54	0.62	2.11	3.09	3.17	Crebbp	3.38
cicRNA.152	1.78	1.32	1.78	2.54	4.10	3.49	Hectd2	3.37
cicRNA.14323	17.04	17.25	17.19	19.21	18.06	19.46	Psap	3.36
cicRNA.9268	0.41	0.36	0.36	1.73	3.21	1.40	Mast4	3.33
mmu_circ_0003562	16.50	16.27	16.15	17.76	18.02	18.34	Luc7l3	3.33
cicRNA.4916	16.31	17.06	16.22	17.62	17.99	19.16	Kif21a	3.32
cicRNA.2735	18.77	19.34	18.59	20.83	19.74	21.32	Gm26917	3.32
cicRNA.6666	1.03	0.31	1.26	2.92	2.75	2.11	Rb1	3.31
mmu_circ_0000788	20.78	20.93	20.93	22.93	21.96	22.93	None	3.31
mmu_circ_0000927	16.33	15.94	15.59	17.52	17.81	17.70	Malat1	3.30
cicRNA.2232	0.38	0.44	0.44	1.75	3.00	1.67	L3mbtl4	3.29
cicRNA.27733	19.74	19.63	19.51	21.68	20.52	21.83	Malat1	3.28
mmu_circ_0002560	18.23	17.68	17.55	19.38	19.73	19.48	Anks1b	3.27
mmu_circ_0004508	1.95	2.07	1.42	2.80	3.73	4.02	Hecw1	3.25
cicRNA.27286	15.60	15.81	15.59	17.36	16.74	17.98	Rtf1	3.23
cicRNA.19455	15.60	15.79	15.48	16.75	17.62	17.47	Aplp2	3.15
cicRNA.23186	15.81	16.09	15.71	17.39	17.69	17.50	Pde4dip	3.14
cicRNA.6639	1.00	0.56	0.63	1.69	3.45	2.01	Rubcnl	3.14
cicRNA.26713	16.24	16.54	16.22	18.16	17.19	18.59	Dstn	3.13
mmu_circ_0003563	15.21	14.84	15.08	16.39	16.69	16.95	Luc7l3	3.11
cicRNA.6049	15.86	15.86	15.76	17.56	16.91	17.93	Mtdh	3.11
cicRNA.5793	16.05	16.06	15.77	18.01	17.39	17.34	Enpp2	3.08
cicRNA.19032	3.98	3.68	4.40	5.77	5.90	5.25	Rfx7	3.07
cicRNA.6263	0.30	0.27	0.27	1.20	1.93	2.56	n/a	3.07
mmu_circ_0000835	3.19	2.58	2.50	3.66	4.84	4.64	None	3.07
cicRNA.27155	15.75	15.46	15.35	17.24	16.13	18.03	Pdia3	3.06
cicRNA.1790	16.67	16.99	16.89	18.72	17.64	19.01	Calm2	3.04
cicRNA.27740	20.13	20.23	19.85	22.02	20.77	22.22	Malat1	3.03
mmu_circ_0010630	0.92	0.78	1.28	2.14	3.56	2.09	Usp13	3.03
mmu_circ_0000787	21.30	21.33	21.11	22.59	23.10	22.83	None	3.02
cicRNA.12896	14.69	14.60	14.80	16.45	17.07	15.33	Ogfod3	3.00
cicRNA.3570	14.56	15.19	14.97	15.93	16.74	16.77	App	2.97
cicRNA.27385	0.97	0.61	1.56	2.58	3.45	1.81	BC052040	2.97
cicRNA.7038	16.11	16.11	16.25	17.38	18.00	17.81	Rpph1	2.97
cicRNA.2872	16.59	16.20	16.42	17.70	18.79	17.42	Brd4	2.96
cicRNA.14322	17.35	17.21	17.28	19.28	18.00	19.27	Psap	2.96
cicRNA.25222	1.64	2.56	1.93	3.10	4.58	3.13	Acvr1	2.95
mmu_circ_0006530	15.14	15.16	14.99	16.68	16.15	17.11	Qk	2.93
mmu_circ_0008351	4.36	4.94	5.14	6.61	5.75	6.73	Dnm3	2.92
cicRNA.6994	16.47	16.33	16.31	17.64	18.36	17.75	Slc7a8	2.92

cicRNA.10538	14.85	14.74	14.98	16.06	16.63	16.50	Klc1	2.92
cicRNA.16116	17.65	17.71	17.54	19.40	19.38	18.69	Abi2	2.87
mmu_circ_0000877	14.77	14.80	14.61	16.27	15.76	16.71	Csnk1a1	2.87
cicRNA.21172	1.80	1.90	1.88	3.22	3.97	2.95	Adgrl3	2.87
cicRNA.647	2.83	1.82	1.63	3.43	3.86	3.54	Zfp236	2.87
cicRNA.14324	15.16	15.23	15.17	16.88	15.89	17.35	Psap	2.86
cicRNA.23429	15.15	15.43	15.31	16.73	16.64	17.07	Sh3d19	2.86
mmu_circ_0012208	15.50	15.54	15.60	17.07	17.29	16.83	Mdh2	2.85
mmu_circ_0016270	15.14	15.11	14.81	16.46	16.51	16.58	Xiap	2.83
mmu_circ_0002961	14.01	14.54	14.17	15.88	14.93	16.40	Spnb2	2.83
cicRNA.27798	14.62	14.88	14.81	15.94	16.44	16.42	Ablim1	2.82
cicRNA.7402	15.46	15.73	15.31	16.82	17.03	17.13	Zmiz1	2.82
mmu_circ_0006380	14.73	14.58	14.61	15.86	16.85	15.70	Rbfox1	2.82
cicRNA.25598	14.12	14.41	14.12	15.51	15.98	15.65	Prrc2b	2.82
cicRNA.3471	16.41	16.31	16.26	17.73	18.33	17.40	Ifnar1	2.81
cicRNA.10537	15.10	15.05	15.13	16.35	16.84	16.55	Klc1	2.80
cicRNA.3648	14.41	14.28	14.30	15.54	16.65	15.25	Rbfox1	2.80
cicRNA.13803	0.38	0.51	0.48	1.94	2.36	1.49	Aldh1l2	2.78
cicRNA.4576	14.96	14.71	14.69	16.04	16.80	15.93	Pdxdc1	2.77
cicRNA.15112	1.87	1.56	0.64	3.37	2.88	2.23	Rassf3	2.77
cicRNA.18434	13.90	14.03	14.10	15.61	14.58	16.24	Lamp2	2.76
deep:mmu-circRNA1119=bartel_circRNA-120	14.94	14.75	14.81	16.07	16.24	16.58	Luc7l3	2.76
cicRNA.15060	14.30	14.57	14.16	15.62	16.01	15.78	Utrn	2.76
cicRNA.12381	14.24	14.63	14.38	15.99	15.14	16.46	Sqstm1	2.72
cicRNA.25984	14.87	14.92	14.52	16.27	15.61	16.75	Dnajc1	2.71
cicRNA.25409	0.28	0.27	0.27	2.06	0.81	2.26	Golga1	2.71
mmu_circ_0016351	14.44	14.42	14.29	15.90	16.00	15.53	Mecp2	2.69
mmu_circ_0016299	13.86	13.70	13.84	15.53	14.69	15.45	Gpc3	2.68
cicRNA.10253	13.76	13.82	13.59	14.97	15.87	14.60	Lamb1	2.68
mmu_circ_0005973	14.85	15.23	14.58	15.78	16.10	17.05	Kif21a	2.68
cicRNA.14438	0.50	0.44	0.44	1.03	2.44	2.17	Dcbld1	2.67
cicRNA.27488	0.86	0.73	0.80	1.40	2.97	2.27	Ano3	2.67
cicRNA.20697	2.55	2.15	2.83	4.07	4.01	3.69	Osbpl3	2.66
cicRNA.27735	13.77	14.03	13.62	15.01	15.21	15.43	Malat1	2.66
cicRNA.27493	0.54	0.57	0.64	1.73	2.10	2.13	n/a	2.65
mmu_circ_0006237	14.09	14.02	13.88	15.24	15.76	15.21	Pdia5	2.64
cicRNA.2197	2.15	2.82	1.21	3.25	3.52	3.49	Dlgap1	2.56
cicRNA.16355	0.87	0.71	1.15	2.72	2.37	1.61	Tpp2	2.50
cicRNA.27088	1.49	1.74	1.26	2.90	2.95	2.38	Cep152	2.38
cicRNA.20408	3.93	3.32	3.37	4.26	5.41	4.66	Brsk2	2.35
cicRNA.12882	1.92	1.36	2.34	2.51	3.18	3.53	Tbcd	2.30

cicRNA.6181	7.70	8.59	7.66	8.82	9.36	9.33	Trio	2.28
cicRNA.14158	3.33	2.37	2.32	3.52	4.33	3.74	Fam13c	2.28
cicRNA.9671	0.29	0.28	0.27	1.68	1.92	0.78	Rad51b	2.27
cicRNA.9365	3.73	5.07	4.28	5.34	5.66	5.51	Cep128	2.21
cicRNA.15968	0.34	0.46	0.46	0.91	2.18	1.60	n/a	2.21
cicRNA.22336	3.39	4.27	3.54	5.02	5.09	4.51	Fktn	2.20
cicRNA.17322	5.13	5.64	5.47	6.24	6.94	6.46	Pappa2	2.20
cicRNA.20340	2.55	2.20	2.67	3.19	3.93	3.70	Nav2	2.20
cicRNA.648	2.73	2.89	3.28	3.71	4.68	3.82	Zfp236	2.15
mmu_circ_0013449	2.37	2.39	2.55	3.42	3.80	3.40	Chn2	2.15
mmu_circ_0009514	1.40	1.51	2.00	0.63	0.39	0.53	Rprd1b	0.46
cicRNA.27912	2.31	2.33	2.41	1.32	1.70	0.59	Sorcs3	0.45
mmu_circ_0005230	1.24	2.21	1.54	0.58	0.44	0.45	Sh2d4b	0.44
cicRNA.22613	1.50	1.45	2.47	0.60	0.55	0.44	Fhad1	0.41
cicRNA.15519	1.09	2.28	1.82	0.42	0.44	0.43	Sntg1	0.41
cicRNA.27201	1.72	1.80	2.83	0.82	0.88	0.60	Ubr1	0.39
mmu_circ_0016419	1.66	1.42	2.59	0.66	0.45	0.42	Zc4h2	0.38
mmu_circ_0000875	1.77	3.07	2.80	1.72	0.99	0.76	Fbn2	0.38
cicRNA.25207	4.14	4.29	3.83	3.02	2.71	2.33	Ccdc148	0.38
mmu_circ_0005548	2.19	3.15	1.96	1.52	0.87	0.59	Cdh10	0.37
cicRNA.20902	3.13	1.94	2.47	1.62	0.65	0.58	Itpr2	0.34
cicRNA.8735	2.85	1.66	2.39	0.78	0.85	0.58	Mboat1	0.34
cicRNA.16654	1.83	2.43	1.81	0.39	0.36	0.57	Rims1	0.33
mmu_circ_0003894	2.17	2.55	2.27	0.57	0.64	0.92	Cog5	0.33
cicRNA.8894	2.16	2.69	3.08	1.83	0.44	0.68	Gli3	0.32
cicRNA.20715	2.60	3.08	3.25	1.55	1.30	1.10	Tpk1	0.32
cicRNA.10042	2.95	3.11	2.60	0.77	0.61	2.25	Nrcam	0.31
cicRNA.1608	1.42	3.15	2.77	1.19	0.45	0.43	Nol4	0.30
cicRNA.5781	1.66	2.66	2.85	0.39	0.87	0.61	Dscc1	0.29
mmu_circ_0005679	5.89	6.02	6.29	4.05	4.22	4.56	Plcxd3	0.29
cicRNA.1832	2.30	2.18	3.18	0.81	0.86	0.60	Srbd1	0.29
cicRNA.18089	2.30	2.53	2.41	0.61	0.62	0.60	Phlpp1	0.29
cicRNA.17800	3.48	2.70	3.11	2.37	0.83	0.67	Eif2d	0.29
mmu_circ_0002952	3.10	2.96	2.83	1.99	0.48	0.60	Eml6	0.26
cicRNA.23673	1.85	2.74	2.81	0.61	0.48	0.48	Slc7a11	0.26
cicRNA.1263	2.92	3.57	1.47	1.20	0.45	0.42	Stk32a	0.26
cicRNA.21644	2.33	2.27	2.55	0.44	0.38	0.40	Cux1	0.25
cicRNA.12509	3.60	3.71	4.18	1.76	1.23	2.47	Gabrb2	0.25
cicRNA.26508	2.06	3.50	3.46	0.97	1.21	0.80	Uqcc1	0.25
cicRNA.15477	3.65	3.57	2.92	2.29	0.87	0.65	Ilkap	0.23
cicRNA.21638	2.70	3.06	3.61	1.91	0.57	0.54	Cyp3a13	0.23
cicRNA.19477	3.15	2.92	3.52	2.29	0.42	0.33	Dpy19l1	0.22
cicRNA.24503	6.14	5.27	4.95	3.87	2.03	3.79	Dennd2c	0.21

mmu_circ_0007973	3.71	3.64	3.36	2.53	0.54	0.90	Gfra1	0.21
mmu_circ_0016223	4.04	2.65	3.02	1.61	0.81	0.54	Ap1s2	0.21
cicRNA.11809	3.56	5.21	5.26	2.17	1.71	3.32	Aurkb	0.21
cicRNA.20245	3.96	4.05	2.74	2.65	0.58	0.54	Rsf1	0.20
cicRNA.17441	3.61	4.39	1.52	0.31	0.46	1.18	Cacna1e	0.17
cicRNA.26231	2.67	5.15	1.99	0.45	0.38	0.43	Cse1l	0.14
cicRNA.1613	3.81	3.99	4.03	1.24	0.54	0.50	Klhl14	0.11
cicRNA.26505	3.70	5.80	4.07	1.89	0.39	0.43	Uqcc1	0.08
